# Sphingosine kinase and sphingosine-1-phosphate regulate epithelial cell architecture by the modulation of *de novo* sphingolipid synthesis

**DOI:** 10.1371/journal.pone.0213917

**Published:** 2019-03-21

**Authors:** Bruno Jaime Santacreu, Lucila Gisele Pescio, Daniela Judith Romero, Gerardo Raúl Corradi, Norma Sterin-Speziale, Nicolás Octavio Favale

**Affiliations:** 1 Universidad de Buenos Aires, Facultad de Farmacia y Bioquímica, Cátedra de Biología Celular y Molecular, Buenos Aires, Argentina; 2 Consejo Nacional de Investigaciones Científicas y Técnicas (CONICET)—Universidad de Buenos Aires, Instituto de Química y Fisicoquímica Biológicas (IQUIFIB), Buenos Aires, Argentina; 3 Universidad de Buenos Aires. Facultad de Farmacia y Bioquímica. Departamento de Química Biológica. Cátedra de Química Biológica Superior, Buenos Aires, Argentina; 4 Consejo Nacional de Investigaciones Científicas y Técnicas (CONICET)—Universidad de Buenos Aires, Instituto de Química y Fisicoquímica Biológicas (IQUIFIB), Laboratorio Nacional de Investigación y Servicios de Péptidos y Proteínas—Espectrometría de Masa (LANAIS PROEM), Buenos Aires, Argentina; Virginia Commonwealth University Medical Center, UNITED STATES

## Abstract

Sphingolipids regulate several aspects of cell behavior and it has been demonstrated that cells adjust their sphingolipid metabolism in response to metabolic needs. Particularly, sphingosine-1-phosphate (S1P), a final product of sphingolipid metabolism, is a potent bioactive lipid involved in the regulation of various cellular processes, including cell proliferation, cell migration, actin cytoskeletal reorganization and cell adhesion. In previous work in rat renal papillae, we showed that sphingosine kinase (SK) expression and S1P levels are developmentally regulated and control *de novo* sphingolipid synthesis. The aim of the present study was to evaluate the participation of SK/S1P pathway in the triggering of cell differentiation by external hypertonicity. We found that hypertonicity evoked a sharp decrease in SK expression, thus activating the *de novo* sphingolipid synthesis pathway. Furthermore, the inhibition of SK activity evoked a relaxation of cell-cell adherens junction (AJ) with accumulation of the AJ complex (E-cadherin/β-catenin/α-catenin) in the Golgi complex, preventing the acquisition of the differentiated cell phenotype. This phenotype alteration was a consequence of a sphingolipid misbalance with an increase in ceramide levels. Moreover, we found that SNAI1 and SNAI2 were located in the cell nucleus with impairment of cell differentiation induced by SK inhibition, a fact that is considered a biochemical marker of epithelial to mesenchymal transition. So, we suggest that the expression and activity of SK1, but not SK2, act as a control system, allowing epithelial cells to synchronize the various branches of sphingolipid metabolism for an adequate cell differentiation program.

## 1. Introduction

Sphingolipids regulate several aspects of cell behavior and it has been demonstrated that cells adjust their sphingolipid metabolism in response to metabolic needs [1,2]. The *de novo* synthesis of sphingolipids begins with the condensation of serine and a fatty acylCoA by serine palmitoyl-CoA transferase (SPT) to form 3-ketosphinganine, followed by its reduction to dihydrosphingosine, to be further acylated to form dihydroceramide (DHCer), which is then desaturated to form ceramide (Cer). Cer is the central core lipid in the metabolism of sphingolipids from which sphingomyelin (SM) and glycosphingolipids are synthesized. Cer is also produced by the salvage pathway, initiated by hydrolysis of SM or glycosphingolipids. Cer can be broken down by ceramidases to form sphingosine, which is in turn phosphorylated by sphingosine kinase (SK) to form sphingosine-1-phosphate (S1P) [1,3,4]. S1P is a final product of sphingolipid metabolism and its degradation by the S1P lyase serves as a single point of degradation of all sphingolipids. S1P is a potent bioactive lipid involved in the regulation of various cellular processes, such as cell proliferation, cell migration, actin cytoskeletal reorganization and cell adhesion [5,6]. As a signaling molecule, S1P exerts effects through both intracellular and extracellular mechanisms [7]. In previous work, we showed that SK/S1P pathway is developmentally regulated in rat renal papillae [8]. Thus, the developmental regulation of SK expression and activity leads sphingolipid metabolism to high levels of S1P in the neonatal period and a decreased expression of SK in the adult. We have also shown that the SK/S1P pathway controls the *de novo* synthesis of sphingolipids, exerting a negative modulation of SPT and DHCer/Cer synthases and displaying a dynamic interplay between the *de novo* synthesis and S1P levels [9].

Madin-Darby Canine Kidney (MDCK) is a cell line derived from dog renal collecting ducts used as a model to study epithelial cell polarization and differentiation [10]. The physiological condition for renal collecting duct cells is external hypertonicity, a condition under which MDCK cells express channels, pumps and co-transporters, typical of functional differentiated cells [11–13]. We have also previously reported that, under external hypertonicity, MDCK cells acquire the fully differentiated morphological epithelial cell phenotype, which is driven by changes in sphingolipid metabolism. In fact, cell differentiation is accompanied by an increase in SM synthesis and an increase in C24:0 and C24:1 glucosylceramide (GluCer) generated from the Cer recycling pathway [14–16]. While the increase in SM synthesis is involved in the assembly of a mature adherens junction (AJ) [15], the development of a differentiated apical membrane domain depends on GluCer synthesis [14].

In epithelia, AJ assembly is essential for the development and maintenance of tissue structure and mandatory for epithelial cell polarization. The most important component of epithelial AJ is E-cadherin (E-cad), which, by homophilic contacts, builds a cluster on the surface of epithelial cells to determine cell shape, maintain tissue integrity, and generate forces throughout the tissue. E-cad maintains cytoskeletal dynamics through linkage of the cytoplasmic domain to the actin cytoskeleton via catenins. While β-catenin (β-cat) binds directly to the distal amino acids of the E-cad cytoplasmic domain, α-catenin (α-cat) associates both with β-cat and the actin cytoskeleton. The stoichiometry of the E-cad/β-cat/α-cat complex is 1:1:1. Previous studies have reported that β-cat binds to the cytoplasmic domain of E-cad shortly after E-cad is synthesized at the endoplasmic reticulum (ER) and that such complex coupling assembly is necessary for an efficient ER exit [17,18]. Contrary to the implication of E-cad as a key molecule in cell differentiation, loss of E-cad expression is often used as a marker for epithelial to mesenchymal transition (EMT) and cancer progression [19,20]. E-cad is potentially repressed by Slug (SNAI2) and Snail (SNAI1), which are master regulatory transcription factors involved in the EMT of cancer cells [21–23].

Considering our previous observation that S1P production is developmentally regulated, showing the highest value in proliferating non-differentiated cells and gradually decreasing to finally reach the lowest level in fully differentiated tissue [9], we were interested in studying whether the production of S1P plays a role in the process of cell differentiation. We found that cell differentiation induced by hypertonicity evokes a sharp decrease in SK expression, thus inducing the regulation of the *de novo* sphingolipid synthesis pathway. Furthermore, the inhibition of SK activity evokes a relaxation of cell-cell AJ with accumulation of the AJ complex (E-cad/β-cat/α-cat) in the Golgi complex, preventing the acquisition of a differentiated phenotype.

## 2. Materials and methods

### 2.1. Cell culture and treatment

MDCK cells (ATCC) were seeded at 0.2 x 10^6^ cells/mL density in six-well multidishes (growth area 9.5 cm^2^) and grown in DMEM/F-12 (42400010, Thermo Fisher Scientific) containing 10% fetal bovine serum (FBS), penicillin (100 μg/mL) and streptomycin (100 μg/mL) at 37° C in humidified 5% CO_2_ atmosphere. After 24 h, the medium was replaced by DMEM/F12 containing 0.5% FBS to synchronize the cell cycle, and cells were incubated for another 24 h. Proliferative cells corresponded to the culture obtained at this point (Prolif). Completely confluent cells were incubated for additional 48 h in the same conditions (Iso). For differentiated cells, proliferative cells were switched to a hypertonic medium (300 mM NaCl) containing 1% of FBS with or without inhibitors and further incubated for 48, 72 or 96 h. S1P-depleted FBS was used as control in a parallel experiment. SK was inhibited by L-*threo*-dihydrosphingosine and SKI II, SPT by myriocin at 100 nM and Cer synthase by Fumonisin B1 at 20 μM (all from Sigma-Aldrich). In the experiment where exogenous S1P was added, the final concentration was 10 μM. S1PR_1_ was blocked by 10 μM W146 (Sigma-Aldrich) and S1PR_2_ by 10 μM JTE-013 (Sigma-Aldrich). The treatments were performed for 48, 72 or 96 h, as indicated in Results. For all the incubations, the medium containing the enzyme inhibitors or the S1P was replaced every 24 h. At the end of each incubation, the cells were collected by trypsinization and counted. Cell viability was determined by the Trypan Blue Exclusion method. To obtain S1P-depleted FBS, 1 volume of FBS was extracted with n-butanol:diisopropyl ether 1:2 according to the method of Cham and Knowles [24]. Two additional steps of solvent evaporation by nitrogen stream for 2 h and a dialysis against PBS in 250 volumes with three buffer exchanges at 4°C for 3 days were performed.

### 2.2. Sphingolipid analysis

In order to evaluate sphingolipid synthesis, MDCK cells were incubated with 25 nCi/mL radioactive precursor [1-^14^C] palmitic acid (PerkinElmer) for 4 h to evaluate newly synthetized metabolites [25]. After trypsinization, lipids were extracted by the method of Bligh and Dyer and mild alkaline hydrolysis was performed for sphingolipid extraction [25]. After extracted lipid were dried by a stream of nitrogen, sphingolipid was resolved by thin layer chromatography (TLC). SM and S1P were resolved by Silica Gel 60 TLC plates (Merck Millipore) using 1-butanol:acetic acid:water (3:1:1, v:v:v). For Cer and GluCer analysis, the TLC was developed in two different solvents. First, the TLC was developed to two-thirds of the plate in the same solvent system as before and then, the plate was cut at Rf = 0.8 (just above the sphingosine standard), and the remaining piece of the plate was developed in chloroform:methanol (50:1.5) [15,16]. Radioactive resolved sphingolipids were scanned (Storm 840, GE Healthcare). The corresponding radioactive spots were scraped off TLC plates for additional measurement by liquid scintillation counting.

### 2.3 Sphingosine kinase activity

SK activity was assessed by measuring the conversion of [^3^H] sphingosine to [^3^H] S1P. Before trypsinization, MDCK cells were incubated for 4 h with 0.6 μCi [3-^3^H] sphingosine (PN: NET1072050UC, PerkinElmer Life Sciences). S1P extraction was optimized by using 0.1 N HCl for phase separation during Bligh and Dyer [26]. Both [^3^H] sphingosine and [^3^H] S1P were resolved and analyzed as described above and visualized with ninhydrin using standard-quality sphingosine and S1P. For endogenous S1P measurement, iodine vapors were used for visualization.

### 2.4. Immunoblot analysis

After washing with phosphate buffer saline (PBS), cells were scraped, resuspended in lysis buffer (50 mM HEPES, 1% Triton X-100, 150 mM NaCl, 1 mM PMSF, 1 μg/mL aprotinin, 1 mM leupeptin, 200 μM NaVO_4_), and sonicated. Aliquots were incubated with 4X Laemmli buffer at 100°C for 5 min and resolved in a 12.5% SDS-polyacrylamide gel and blotted to polyvinylidene fluoride membranes. Blots were blocked with 5% nonfat milk in TBS-Tween incubated overnight at 4°C with 1:1000 goat antibody anti-SK1 (polyclonal, PC727; Oncogene) in 1% BSA in TBS-Tween. After washing, blots were incubated with donkey 1:5000 anti-goat antibodies (polyclonal, W10825; Thermo Fisher Scientific) and bands evidenced by means of the ECL plus analysis system (32106; Thermo Fisher Scientific). The intensity of each band was estimated by optical densitometry by using ImageJ software.

### 2.5. Fluorescence microscopy

MDCK cells were grown on coverslips during cell seeding. At the end of the corresponding treatments cells were washed with PBS and fixed with 4% (m/v) paraformaldehyde at 25° C for 20 min, and permeabilized with 0.1% (v/v) Triton X-100 at 25° C for 20 min. After washing with PBS, cells were incubated with 3% BSA in PBS at 25° C for 60 min, followed by incubation for 90 min with the primary antibodies: 1:250 mouse anti-α-catenin (polyclonal, C2081; Sigma), 1:200 rabbit anti-β-catenin (monoclonal, C7082; Sigma), 1:50 rabbit anti-E-cadherin (polyclonal, SC-7870, Cruz Biotechnology), 1:300 mouse anti-giantin (monoclonal, ab37266; Abcam), 1:50 rabbit anti-SNAI1 (polyclonal, SC-281999; Santa Cruz Biotechnology) and 1:50 rabbit anti-SNAI2 (polyclonal, SC-15391; Santa Cruz Biotechnology). Primary interactions were detected by using a 1:200 F(ab ´)_2_ fragment of goat anti-rabbit IgG and a 1:200 F(ab ´)_2_ fragment of goat anti-mouse IgG (polyclonal, 115-095-166, 115-024-166, 111-025-144, 111-095-003; Jackson ImmunoResearch), both FITC or TRITC. Actin filaments were visualized by 1 μg/mL phalloidin-FITC (P5282, Sigma-Aldrich) and the ER with 0.20 μg/mL concanavalin A-TRITC (C860; Thermo Fisher Scientific). The coverslips were then mounted onto microscope glass slides with Vectashield Mounting Media (H-1000; Vector Laboratories) and stored at -20°C until analysis.

### 2.6. Cell transfection

MDCK cells grown on coverslips were subjected to a transient protein expression protocol. Transfection was carried out 4 h before adding the hypertonic medium by using 0.5 μg of plasmid and the TransIT-X2 Dynamic Transfection System (MIR6003, Mirus Bio LLC), according to the manufacturer's instructions. GalT2-YFP plasmid was a gift from Dr. Daniotti [27].

For siRNA transfection, MDCK cells were grown in DMEM/F-12 containing 10% FBS without antibiotics for 24 h. Then, cells were cultured in DMEM/F-12 containing 0.5% FBS and transfected with 100 nM double-stranded siRNAs for SK1, SK2 or negative sequence using HiPerFect transfection reagent (301705, Qiagen) according to the manufacturer’s instructions. After 24 h, transfection reagents were washed out and the medium was replaced by DMEM/F-12 containing 1% FBS and incubated with 300 mM NaCl for 48 h. The sequences of siRNAs for SK1 were 5’ GGGCAAGGCUCUGCAGCUCdTdT 3’ (sense) and 5′ GAGCUGCAGAGCCUUGCCCdTdT 3′ (antisense); for SK2 were 5’ GGGCAAGGCUCUGCAGCUCdTdT 3’ (sense) and 5’ UUCAGCUCCUUAUCGGCGCdTdT 3’ (antisense); and the sequences for the negative siRNA were 5′ CAGUCGCGUUUGCGACUGG 3′ (sense) and 5′ CCAGUCGCAAACGCGACUG 3′ (antisense). To identify the siRNA transfected cells during the fluorescent microscopy analysis, cells were co-transfected with Negative Control siRNA-Alexa546 (Qiagen) according to the manufacturer’s protocol.

### 2.7. Imaging and data processing

Cells were examined by confocal immunofluorescence using an Olympus FV300 confocal microscope (model BX61) equipped with Ar and He-Ne lasers, and 60× oil immersion and numerical aperture 1.40. Images were taken with the acquisition software FluoView version 3.3. A minimum of 10 fields containing several cells were collected from each sample. Manders' overlap coefficient was obtained using Coste’s auto-thresholding in ImageJ software. The line profile was performed in the corresponding area of each cell and data for the graph were obtained using ImageJ software. Nyquist criterion was applied to optimize the lateral (XY) and axial resolution (Z) [28]. Therefore, the microscope was set for a lateral resolution of 46 nm and an axial resolution of 150 nm. Image stacks were processed by ImageJ software for z-plane reconstruction or by UCSF Chimera software for 3D reconstruction. The nucleus/cytoplasm ratio was obtained by creating regions of interest on the Hoechst-positive pixels using the ImageJ software.

### 2.8 Statistics

At least three independent experiments were performed and results were analyzed using Graph Pad Prism Version 6.0 software (Graph Pad Software Inc). Data were shown as the mean ± standard deviation (SD). Statistical comparisons were made by analysis of variance (ANOVA) with Bonferroni post hoc analysis. A ‘p’ value under 0.05 was considered statistically significant.

## 3. Results

### 3.1. Hypertonicity induces changes in SK expression and S1P levels

**Fig 1:**
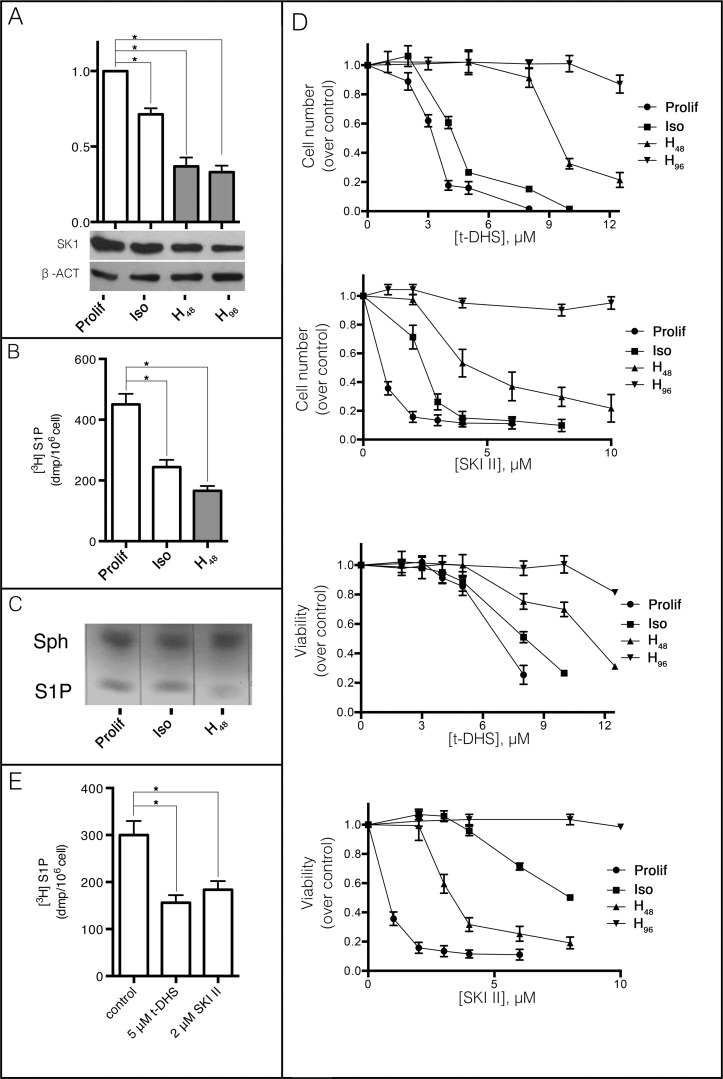
Decrease in SK expression and activity during MDCK cell differentiation. (A) SK protein levels in proliferative (Prolif), polarized (Iso) and differentiated for 48 (H_48_) or 96 h (H_96_) MDCK cells. β-actin was used as endogenous control. (B) SK activity was measured using [3-^3^H] sphingosine as substrate. Prolif, Iso or H_48_ MDCK cells were incubated with 0.6 μCi [3-^3^H] sphingosine for 4 h. Radioactive spots corresponding to [^3^H] S1P on the TLC were scraped and quantified as described under Materials and Methods. (C) S1P from sphingolipid extract derived from Prolif, Iso and H_48_ cells was resolved by TLC and visualized by iodine staining. (D) Cell number and viability were obtained after 48 h in continued SK inhibition conditions with t-DHS or SKI II in Prolif, Iso, H_48_ and H_96_. (E) During MDCK hypertonicity-induced cell differentiation, SK activity was determined by incubation with [^3^H] sphingosine in the presence or absence of SK inhibitors (t-DHS, SKI II). Data are given as mean ± SD with n = 3, *p < 0.05.

Considering that, as we have previously demonstrated, SK is developmentally regulated in rat renal papillae [8] and that external hypertonicity evokes MDCK differentiation [14,15], we first evaluated SK expression in four different culture conditions: cells cultured at 30% confluence (Prolif), cells at 100% confluence under isotonicity (Iso), and 100% confluent cells subjected to hypertonicity for 48 h (H_48_) or 96 h (H_96_), which reflect proliferating, polarized and differentiated cells by hypertonic media for 48 or 96 h, respectively (Fig 1A). As shown in the graph and considering SK expression under proliferative conditions as 100%, we observed a 33% decrease in SK expression in polarized cells and a 65% decrease in differentiated cells (48 h (H_48_) or 96 h (H_96_)). To determine whether differences in protein levels caused differences in SK activity, we further studied S1P production. For this purpose, proliferative (Prolif), polarized (Iso) and differentiated cells (H_48_), were incubated in the presence of [^3^H] sphingosine as substrate and radiolabeling was carried out for 4 h. Then, supernatants were collected and cells were trypsinized and subjected to lipid extraction, followed by methanolysis of phospholipids and triglycerides. Sphingolipids were resolved by TLC as described in Materials and Methods and [^3^H] S1P spots were scraped and counted by Liquid Scintillation Counter. As shown in Fig 1B, the highest production of [^3^H] S1P was obtained in proliferative cells, a 46% decrease was observed in polarized cells and as little as 37% of the control value was obtained in differentiated cells. The same pattern was found when endogenous levels of S1P were analyzed (Fig 1C). Proliferating cells showed the highest levels of S1P, followed by polarized cells, whereas the differentiated cells showed the lowest levels. Summarizing, the differentiated cells presented the lowest expression of SK and S1P synthesis, while proliferating cells showed the highest value of SK expression and S1P production, both consistent with the endogenous amount of S1P found. These results suggest that SK and S1P levels decreased during MDCK cell differentiation.

Previous studies of our laboratory demonstrated that the survival of proliferating MDCK cells is extremely sensitive to S1P synthesis [9]. Thus, we further evaluated S1P dependence of MDCK cell survival by using two different SK inhibitors: t-DHS, a competitive inhibitor of SK, and SKI II, which causes SK degradation [29,30]. Cell viability was analyzed after inhibition of SK for 48 h in proliferating cells, polarized cells, and cells subjected to hypertonicity for 48 or 96 h. The incubation with SK inhibitors was performed in the last 48 h of culture. As during the incubation with hypertonicity for 48 h the cells are in progress to differentiation, the new experimental condition with cells that were subjected to hypertonicity for 48 h and incubated for extra 48 h under hypertonicity in the presence of SK inhibitors (H_96_) was included to evaluate the effect of SK inhibitors in already differentiated cells. The sensitivity of the cells to t-DHS and SKI II decreased as the degree of cell differentiation increased, with proliferating cells being the most sensitive and differentiated cells (H_96_) remaining refractory to SK inhibition (Fig 1D). These results suggest that S1P lost its function of maintaining cell viability as cell differentiation progressed.

Since we were interested in determining the role of SK/S1P pathway in the progress of differentiation, we determined S1P synthesis in cells subjected to hypertonicity (short term incubation) at concentrations of inhibitors that did not affect their survival. The inhibitor concentrations tested were 5 μM for t-DHS and 2 μM for SKI II. [^3^H] sphingosine was incorporated for 4 h after 30 min of hypertonicity with or without inhibitors. Interestingly, MDCK cells in progress to differentiation resisted 51% and 39% inhibition of S1P synthesis with t-DHS and SKI II, respectively (Fig 1E). These results showed that SK inhibitors already decreased S1P synthesis without affecting cell survival.

### 3.2 Inhibition of S1P synthesis disrupts epithelial cell organization

**Fig 2:**
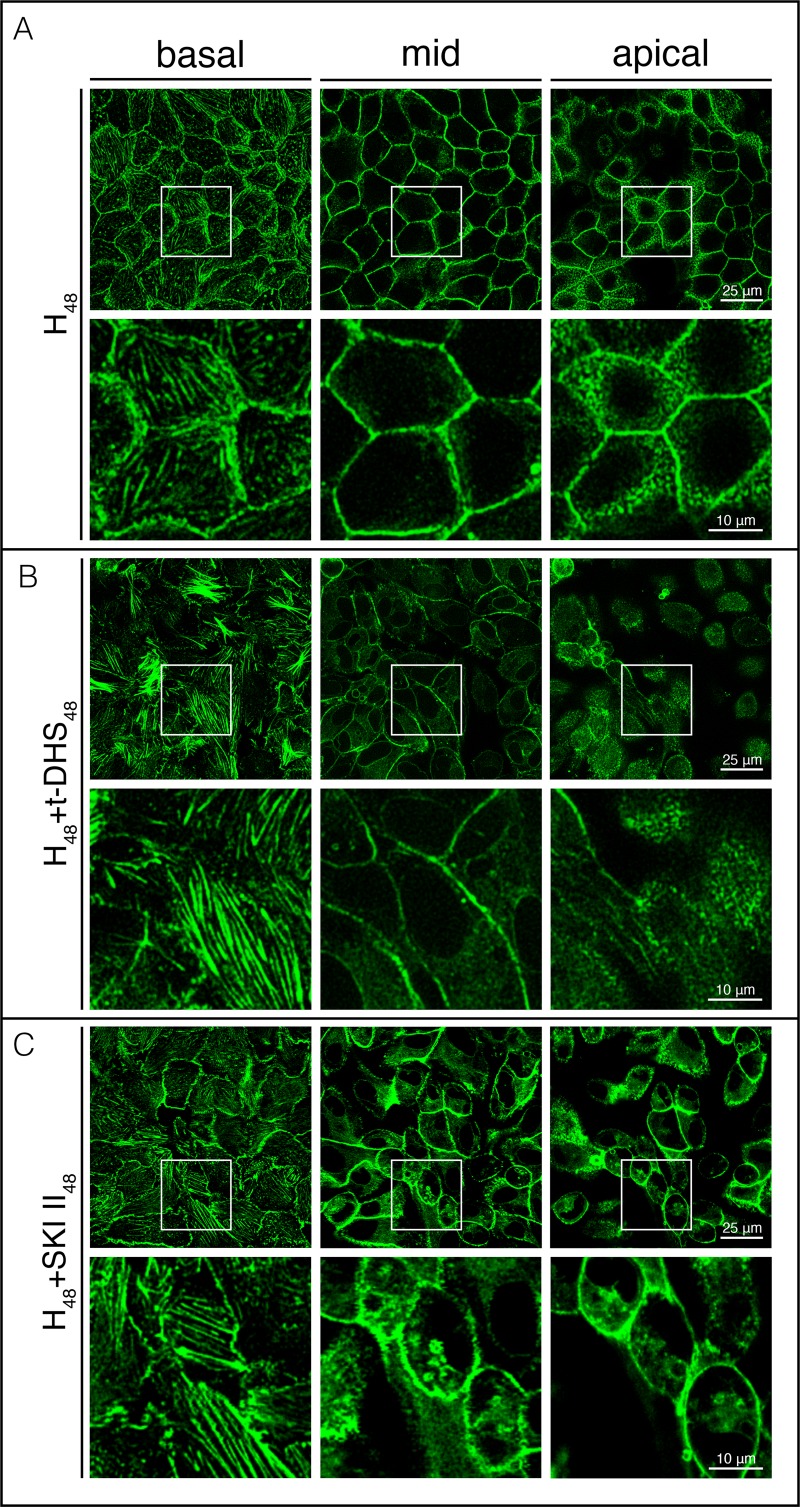
SK inhibition induces actin cytoskeleton reorganization. During MDCK cell differentiation by hypertonicity (Control: Hyper) (A), monolayers were subjected to SK inhibition by t-DHS (B) or SKI II (C). Fixed-cell monolayers were labeled for F-actin with phalloidin-FITC and images were taken by confocal microscopy in 4-μm sections, determining basal, middle (mid) and apical cell planes. High magnification insets of the open rectangles are shown below each image.

After demonstrating that S1P synthesis and SK expression depend on cell physiological conditions, cultured cells subjected to hypertonicity for 48 h were used to explore the influence of S1P synthesis during epithelial cell differentiation and tissue organization. Cell morphology and tissue organization were evaluated by determining F-actin distribution using phalloidin-FITC as fluorochrome and performing confocal microscopy observing three different confocal planes: the most basal plane, where cells are attached to the extracellular matrix, a middle plane, and the most apical plane, which corresponds to the luminal side of the cell. In control cells subjected to hypertonicity for 48 h (H_48_), F-actin appeared delimiting the cells in the three confocal planes analyzed (Fig 2A). In the most basal plane, F-actin was also organized as short stress fibers, thus demonstrating a correct cell-extracellular matrix attachment, while, in the middle plane, F-actin appeared exclusively forming the cellular cortex. In the apical plane, besides drawing the limits of the cells, F-actin showed a punctate appearance, demonstrating the presence of bundles of actin filaments organized into the microvilli typical of the mature apical membrane domain [31]. Next, we examined the effect of blocking S1P production at the time of triggering cell differentiation by hypertonicity by using both inhibitors at concentrations that did not affect the cell number or survival, as described above. Both inhibitors induced alterations in F-actin organization (Fig 2B and 2C). Thus, the cortex of F-actin drawing the cell limits was impaired in the three confocal planes analyzed. Besides the alteration in the cortical F-actin organization, the most basal plane exhibited the formation of longer and parallel fibers of actin, distinctive of migrating and not stabilized epithelial cells. In the middle plane, intracellular puncta of actin were also present, which were more evident in the apical plane. Besides the alteration in F-actin evoked by inhibition of S1P production both with t-DHS and SKI II, the cells subjected to hypertonicity did not acquire the classic hexagonal shape proper of differentiated epithelial cells. Instead, SK inhibition evoked a fibroblast-like phenotype when treated with t-DHS, as observed in the magnified image of the middle plane. In both cases, the cell-cell contact was profoundly altered and, when treated with SKI II, the cells appeared rounded.

### 3.3. S1P biosynthesis is required for mature AJ complex formation

**Fig 3:**
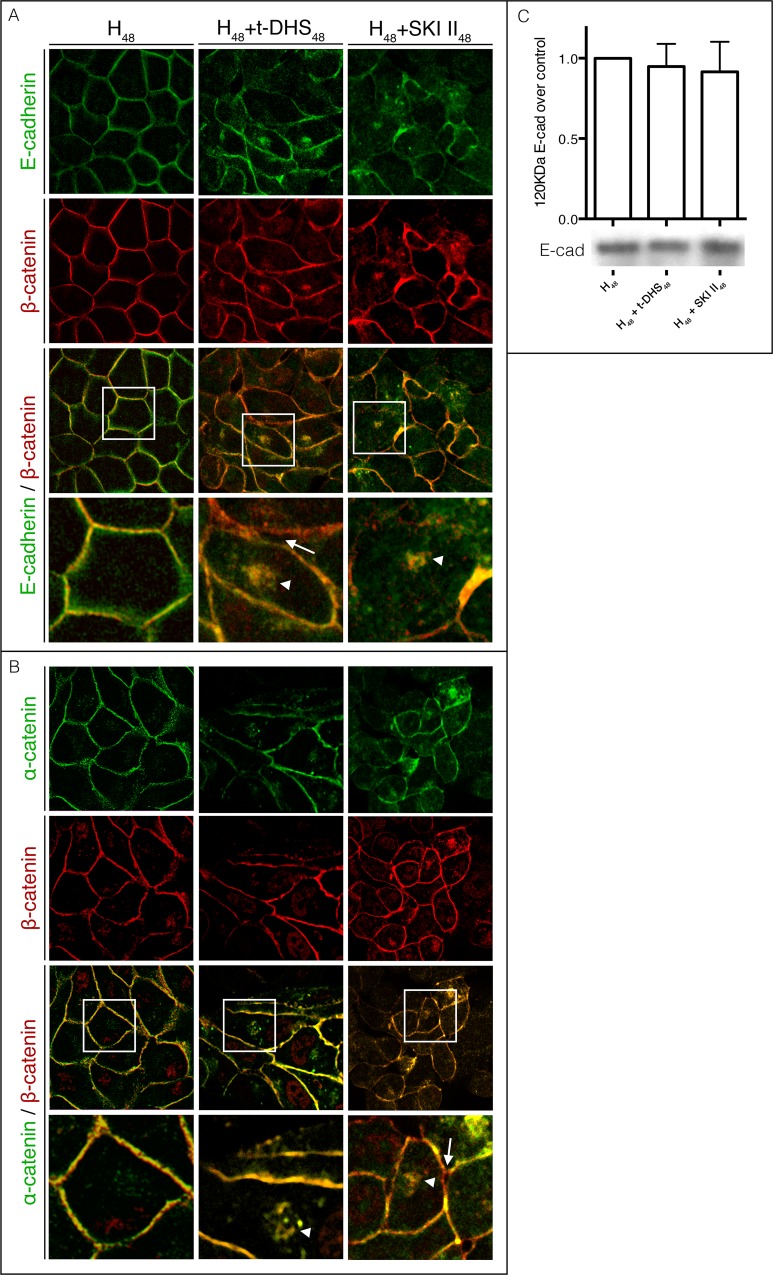
SK inhibition during cell differentiation causes AJ alterations. During MDCK cell differentiation by hypertonicity, monolayers were submitted to SK inhibition (t-DHS or SKI II). Middle confocal planes of monolayers labeled for (A) E-cad (green) and β-cat (red) or (B) α-cat (green) and β-cat (red) and the corresponding merge are shown. High magnification insets of the open rectangles in merge images are shown below each panel. (C) E-cad protein level was determined by Western Blot in differentiated cells (H_48_), or cells treated with t-DHS or SKI (H_48_+t-DHS_48_ or H_48_+SKI II_48_). White arrows indicate zones where cell-cell junction appears separated. White arrowheads indicate intracellular accumulation of AJ complex proteins. The pre-treatment with t-DHS or SKI II caused a deterioration of AJs, evidenced by the appearance of zones where cell-cell separation (arrow) and loss of E-cad and β-cat colocalization accompanied by dissipation from the cell limits were evident, especially in SKI II-treated cells. It is interesting to note the presence of intracellular accumulation of colocalized E-cad and β-cat when cells were treated with the SK inhibitors (arrowhead). Fig 3B shows the localization of α-cat in relationship with β-cat. As before, in differentiated MDCK cells, both proteins colocalized in the periphery of the cells, but the inhibition of SK by t-DHS or SKI II impaired the cell-cell contact denoted by zones where cells lost their contacts and some cells appeared completely separated (arrow), which is more evident in the magnified images. Again, α-cat appeared intracellularly accumulated, colocalizing with β-cat (arrowhead). Taken together, these results demonstrate that the E-cad, β-cat and α-cat complex delocalized from the cell-cell contact and accumulated intracellularly when SK activity was blocked, with the consequent impairment of cell-cell adhesion. Interestingly, although there was a change in the distribution of E-cad, the immunoblot showed no changes between control cells (H_48_) and cells treated with t-DHS or SKI II (H_48_+t-DHS_48_ or H_48_+SKI II_48_) (Fig 3C).

It is accepted that, to organize as epithelial tissue, epithelial cells have to adhere to each other through the formation of stable AJ structures. AJs are visualized by analyzing the distribution of AJ proteins. To assess whether S1P is involved in the acquisition of mature AJs, we first analyzed E-cad and β-cat colocalization and then β-cat and α-cat localization by confocal microscopy. In control differentiated cells (H_48_), the immunolabeling for both E-cad and β-cat showed that they appeared delineating the cell periphery, showing a high degree of colocalization as seen in the merge image, thus reflecting the presence of mature AJs (Fig 3A).

### 3.4. SK inhibition causes accumulation of the AJ protein complex in the *trans-*Golgi network (TGN)

**Fig 4:**
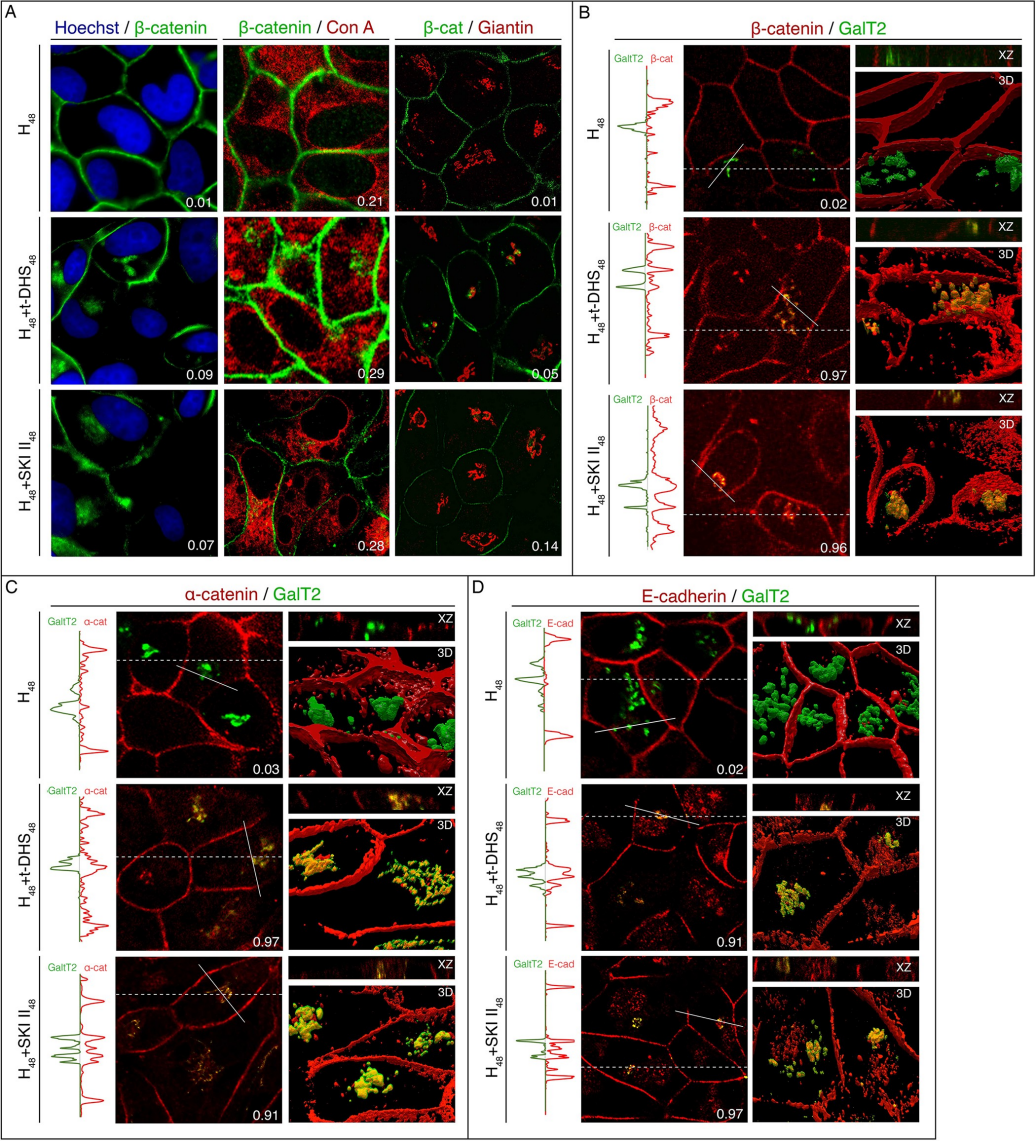
SK inhibition causes accumulation of the AJ protein complex in the TGN. Optical confocal sections perpendicular to the apico-basal axis of the monolayer showing β-cat, nucleus, endoplasmic reticulum and Golgi markers. Dual-color immunofluorescence colocalization was performed on MDCK cells grown on glass coverslips and treated with t-DHS or SKI II. (A) Cells were stained with anti-β-cat (green), and Hoechst for the nucleus (blue), concanavalin A for the endoplasmic reticulum (red) or anti-giantin for the cis/medial-Golgi (red). (B, C, D) MDCK cells transfected with GalT2-YFP (TGN marker) were treated with t-DHS or SKI II for 48 h, fixed and labeled in red for β-cat (B), α-cat (C) or E-cad (D). To the right of each panel, a XZ plane (dotted line in XY plane images) and 3D reconstruction are shown. To the left of each panel, line profiles are displayed in vertical format (solid line in XY plane images). (A-D) Manders’ coefficients (m_1_) are shown in the right inferior angle of each XY image.

After observing that SK inhibition evoked an intracellular accumulation of the AJ complex, we further studied its subcellular localization by confocal microscopy. Colocalization was analyzed by the Manders' Overlap Coefficient with Costes auto-thresholding (MOC), which indicates co-occurring positive pixels of a pair of two fluorophores as a fraction of the total number of positive pixels of each fluorophore (m_1_ and m_2_). The m_1_ and m_2_ values range from 0 to 1.0, where an m_1_ value of 0 means that there are no fluorochrome 1 pixels overlapping with positive pixels for fluorochrome 2, and a value of 1.0 indicates complete overlapping of fluorochrome 1 with positive pixels for fluorochrome 2 [32]. For this set of images, we assigned the AJ proteins pixels in the probed organelle marker pixels as an m_1_ value. Considering that it is accepted that β-cat can translocate to the nucleus and thus operate as a transcription factor, we first investigated the localization of β-cat in the nucleus. To this end, control cells (H_48_) and t-DHS- or SKI II-treated cells were stained by immunofluorescence for β-cat in the presence of a nuclear marker (Hoechst). Both markers had different distribution and β-cat accumulated in the proximity but not in the nucleus (Fig 4A). The quantitative analysis showed that the fractions of β-cat in nuclear positive pixels were m_1_ = 0.01 for control cells (H_48_), m_1_ = 0.09 for t-DHS-treated cells and m_1_ = 0.07 for SKI II-treated cells, thus demonstrating no nuclear accumulation of β-cat.

Then, we tested the colocalization of β-cat with markers for the ER, cis/medial Golgi or TGN. ER visualization was performed by the lectin Concavalin A-TRITC. Both control cells (H_48_) and treated cells (H_48_+t-DHS_48_ or H_48_+SKI II_48_) showed a low level of overlapping between intracellularly accumulated β-cat and concanavalin A-positive pixels (m_1_ = 0.21 for H_48_, m_1_ = 0.29 for t-DHS-treated cells and m_1_ = 0.28 for SKI II-treated cells) (Fig 4A). Thereafter, the cis/medial Golgi was immunostained for giantin and the quantitative overlapping was analyzed (Fig 4A). For both inhibitors, images showed an intense labeling for β-cat in the proximity of the label for giantin, but no colocalization. Thus, the values observed by the colocalization analysis were m_1_ = 0.01 for control cells (H_48_), m_1_ = 0.05 for t-DHS-treated cells and m_1_ = 0.014 for SKI II-treated cells. Finally, the TGN was visualized by detection of GalT2-YFP (UDP-Gal:GA2/GM2/GD2 β-1,3-galactosyltransferase) expression (Fig 4B). Both the confocal images and the MOC showed high colocalization for β-cat and the TGN marker in t-DHS- and SKI II-treated cells, being m_1_ = 0.97 and m_1_ = 0.96, respectively.

We further performed a line profile analysis where a set of intensity values taken from regularly spaced points along a line segment are shown in a graph. The line profile analysis is useful to show colocalization as overlapping peaks. In Fig 4B–4D, line profiles are displayed in vertical format on the left of each set of images corresponding to the solid white line in the microscopy images. The line profile of control cells (H_48_) shows β-cat (to the right side of the graph) only as two peaks located at the AJ area. Instead, pixels positive for GalT2 (to the left side of the graph) are located between the β-cat-positive peaks traced over the image shown. This demonstrates that both signals are differently distributed in the cells. The images obtained for SK inhibition (H_48_+t-DHS_48_ or H_48_+SKI II_48_) show β-cat peaks in the zone of AJs but also a central accumulation with the maximum of this peak occurring at the same points where peaks for GalT2 are located, thus reflecting perfect β-cat-GalT2 co-distribution. Due to the three-dimensional architecture of the Golgi complex, we decided to incorporate additional approaches by reconstructing a lateral view (XZ planes, indicated as a white dotted line) and a 3D view. To this end, Z-series of confocal images were collected every 0.15 μm calculated based on Nyquist criterion [28] from monolayers subjected to hypertonicity in the presence or absence of SK inhibitors. As seen in the images, the ZX and 3D plane reconstruction show that, in control cells (H_48_), β-cat is present only as a line delimiting the cells while GalT2 is intracellular, but that, in treated cells, β-cat appears as an underdrawing line and intracellularly colocalizing with GalT2.

Thereafter, we also analyzed the α-cat and E-cad accumulation induced by SK inhibition in the TGN. To this end, cells were immunostained for α-cat (Fig 4C) or E-cad (Fig 4D) in combination of GalT2-YFP expression. The images, line profile, reconstructions and MOC confirmed the co-distribution of both α-cat and E-cad with the TGN marker, showing a perfect coincidence between the intracellular localization of AJ protein and GalT2-YFP.

Taken together, these results and those in Fig 3 suggest that E-cad, β-cat and α-cat accumulate as a complex in the TGN when SK is inhibited in cells in progress to differentiation.

### 3.5. The morphological changes evoked by the SK inhibitors are due to an increase in sphingolipid synthesis

We have previously demonstrated that endogenously generated S1P negatively modulates SPT and DHCer sintase (DHCerS) in proliferating cells, thus controlling the *de novo* synthesis of sphingolipids [9]. To evaluate the possible implication of sphingolipid metabolism in the alteration of cell-cell contact evoked by SK inhibition, we tested the combined effect of Myr or Fumo, inhibitors of SPT and DHCerS/CerS, respectively, and the SK inhibitors, in the acquisition of the differentiated phenotype of MDCK cells. To this end, phalloidin-FITC was used to visualize the actin cytoskeleton and immunofluorescence for β-cat was used to detect AJs.

**Fig 5:**
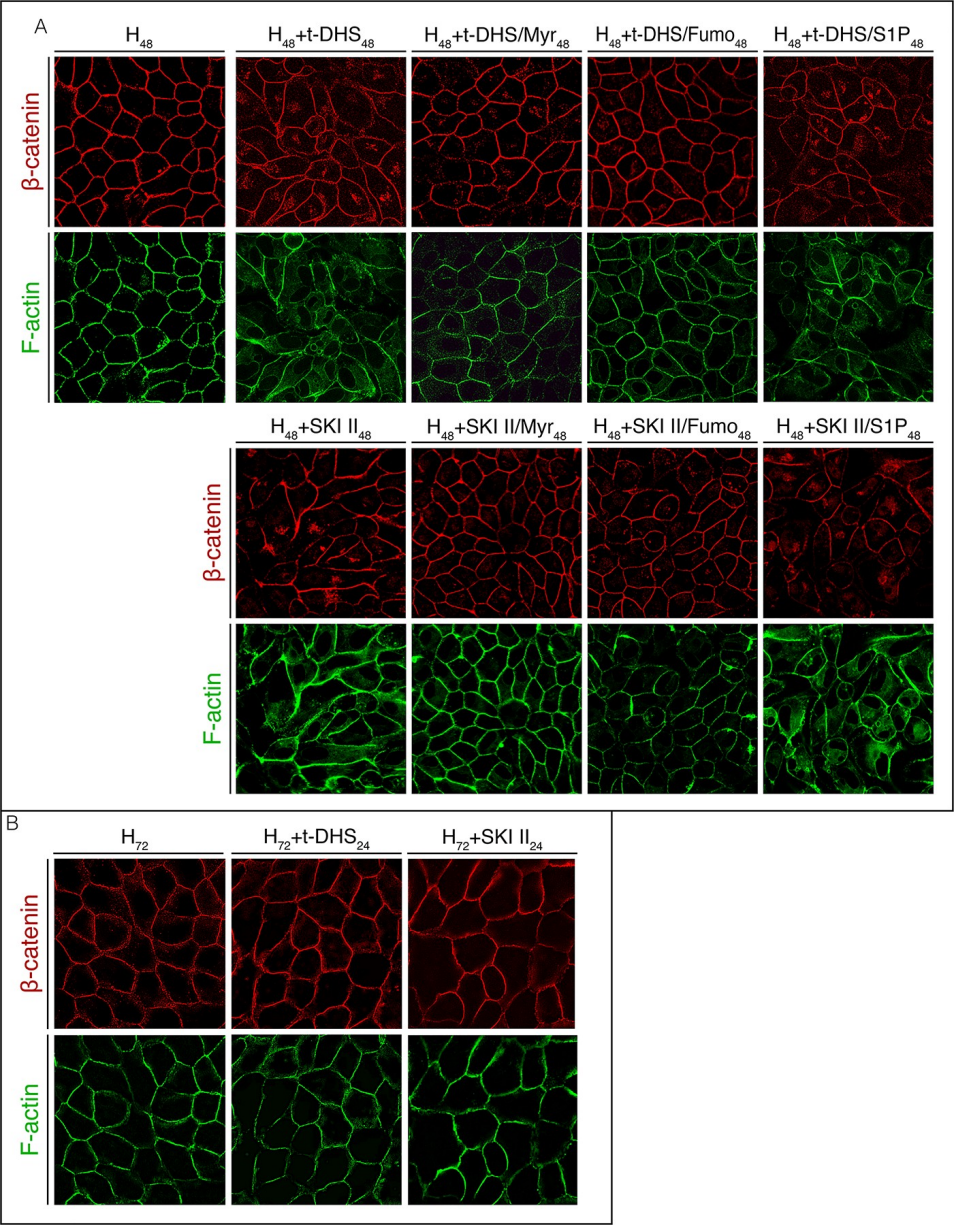
Blocking *de novo* sphingolipid synthesis protects from the impact of SK inhibition. The acquisition of the differentiated phenotype was visualized. (A) SK inhibition (H_48_+t-DHS_48_ or H_48_+SKI II_48_) was combined with Myr, Fumo (H_48_+t-DHS/Myr_48_ or H_48_+SKI II/Fumo_48_) or exogenous S1P(H_48_+t-DHS/S1P_48_ or H_48_+SKI II/S1P_48_). (B) After 48 h of hypertonicity, cells were treated with t-DHS or SKI II for 24 additional hours in hypertonic medium (H_72_, H_72_+t-DHS_24_, H_72_+SKI II_24_).

Treatment with Myr or Fumo over SK inhibition prevented the effect of SK inhibition and cells preserved the typical epithelial cell morphology, reflected by the preservation of the F-actin cortex (Fig 5A). Moreover, β-cat remained delimiting the cell periphery and only slightly stained for the intracellular accumulation. Since S1P is an intracellular mediator but can also act extracellularly, we further added exogenous S1P over SK inhibition and observed the distribution of F-actin and β-cat. The addition of S1P was not able to rescue the cell phenotype even after 48 h of extracellular hypertonicity in the presence of exogenous S1P (Fig 5A). Besides the alteration in cell morphology, dissipation of β-cat from the cell limits with its intracellular accumulation was also observed. Since FBS contains S1P, we used S1P-depleted FBS at the moment of inducing cell differentiation by external hypertonicity to rule out S1P influence in the cultured cells. Results showed non-significant differences in β-cat or F-actin distribution between cells cultured in S1P-depleted and non-depleted medium (S1 Fig).

These results suggest that sphingolipid metabolism is involved in the deleterious effect of SK inhibition and that endogenous synthesis of S1P is required for the acquisition of the epithelial phenotype in MDCK cells subjected to external hypertonicity.

The above results suggest that endogenous synthesis of S1P is required for the acquisition of the typical epithelial phenotype of MDCK cells. Thereafter, we were interested in determining whether endogenous S1P is also necessary for the maintenance of the differentiated phenotype. To this end, we incubated cells for 48 h under hypertonicity to reach a differentiated state and then inhibited SK activity for additional 24 h. Fluorescence microscopy of β-cat and F-actin (Fig 5B) showed that SK inhibition did not cause changes in cell morphology, reflected by F-actin distribution. β-cat remained delimiting the cell periphery without intracellular accumulation. These results demonstrate that MDCK cells in progress to differentiation are sensitive to SK inhibition while endogenous S1P does not play a role in the maintenance of the differentiated state.

### 3.6. SNAI1 and SNAI2 are involved in the impairment of cell differentiation by SK inhibition

**Fig 6:**
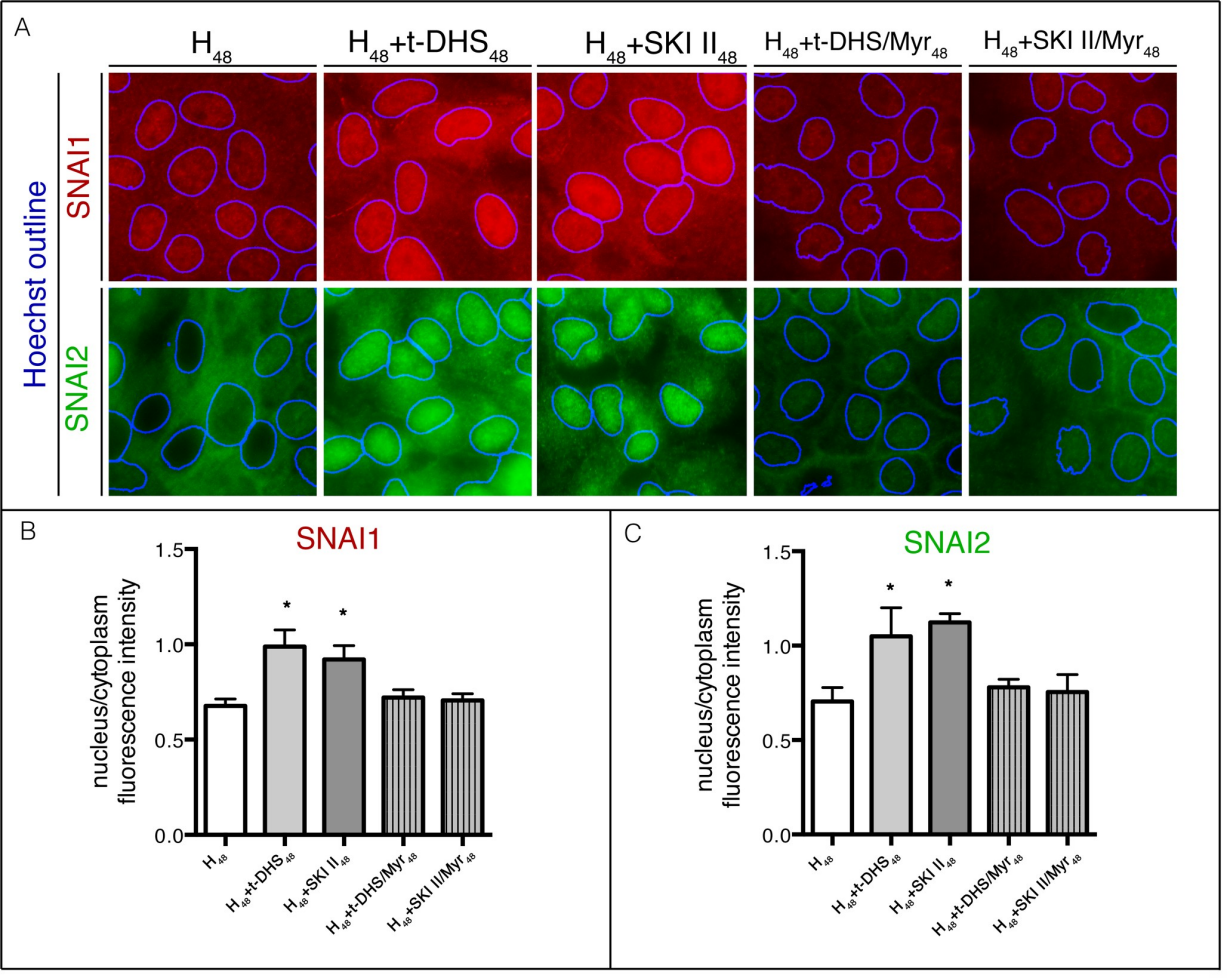
Endogenous SNAI1 and SNAI2 are distributed in the nucleus of SK-inhibited cells. (A) MDCK cells under hypertonicity (H_48_) in the presence of t-DHS, SKI II and t-DHS or SKI II in combination with Myr were labeled for SNAI1 (red) or SNAI2 (green) and the nucleus by Hoechst. Nuclei are shown as outlines of the Hoechst-positive pixels. Quantification of SNAI1 (B) or SNAI2. (C) Fluorescence intensity quantification is shown as the nucleus/cytoplasm ratio. Data are given as mean ± SD with n = 3, *p< 0.05.

The previous result showed that, when MDCK cells were subjected to hypertonicity in the presence of SK inhibitors, instead of acquiring an epithelial phenotype, they adopted a fibroblast-like morphology accompanied by deterioration of AJs. Since it is accepted that both phenomena can be related to an EMT, we further evaluated the expression and localization of the transcription factors SNAI1 (Snail) and SNAI2 (Slug), whose nuclear localization is considered a biochemical marker of EMT [21,33–35]. To this end, H_48_ and t-DHS- and SKI II-treated cells were immunolabeled for SNAI1 and SNAI2 detection. Hoechst labeling was used to digitally determine the nuclei outlines (Fig 6A). Control cells (H_48_) showed neither SNAI1 nor SNAI2 nuclear location. In contrast, SK inhibition by both inhibitors induced nuclear accumulation of SNAI1 and SNAI2. To evaluate the involvement of sphingolipid metabolism in the nuclear translocation of SNAI1/2, over the inhibition of SK, we also inhibited SPT with Myr.

The immunofluorescence images show that the inhibition of sphingolipid metabolism prevented the nuclear translocation of SNAI1/2, thus reflecting involvement of sphingolipid synthesis in the process. The quantitative analysis of the immunofluorescence showed that the nucleus/cytoplasm ratio for SNAI1 was 0.67 in control cells (H_48_) while those for t-DHS and SKI II were 0.98 and 0.92, respectively. The quantification of the ratio for SNAI2 showed the same pattern, resulting in 0.70 for control (H_48_), 1.05 for t-DHS and 1.12 for SKI II. Taken together, these results demonstrate the involvement of sphingolipid metabolism in the nuclear translocation of SNAI1 and 2 induced by SK inhibition. The inhibition of sphingolipid metabolism in SK-inhibited cells by Myr restored the nucleus/cytoplasm ratio to control values (Fig 6B and 6C).

### 3.7. Sphingolipid metabolism is increased in cells treated with SK inhibitors

**Fig 7:**
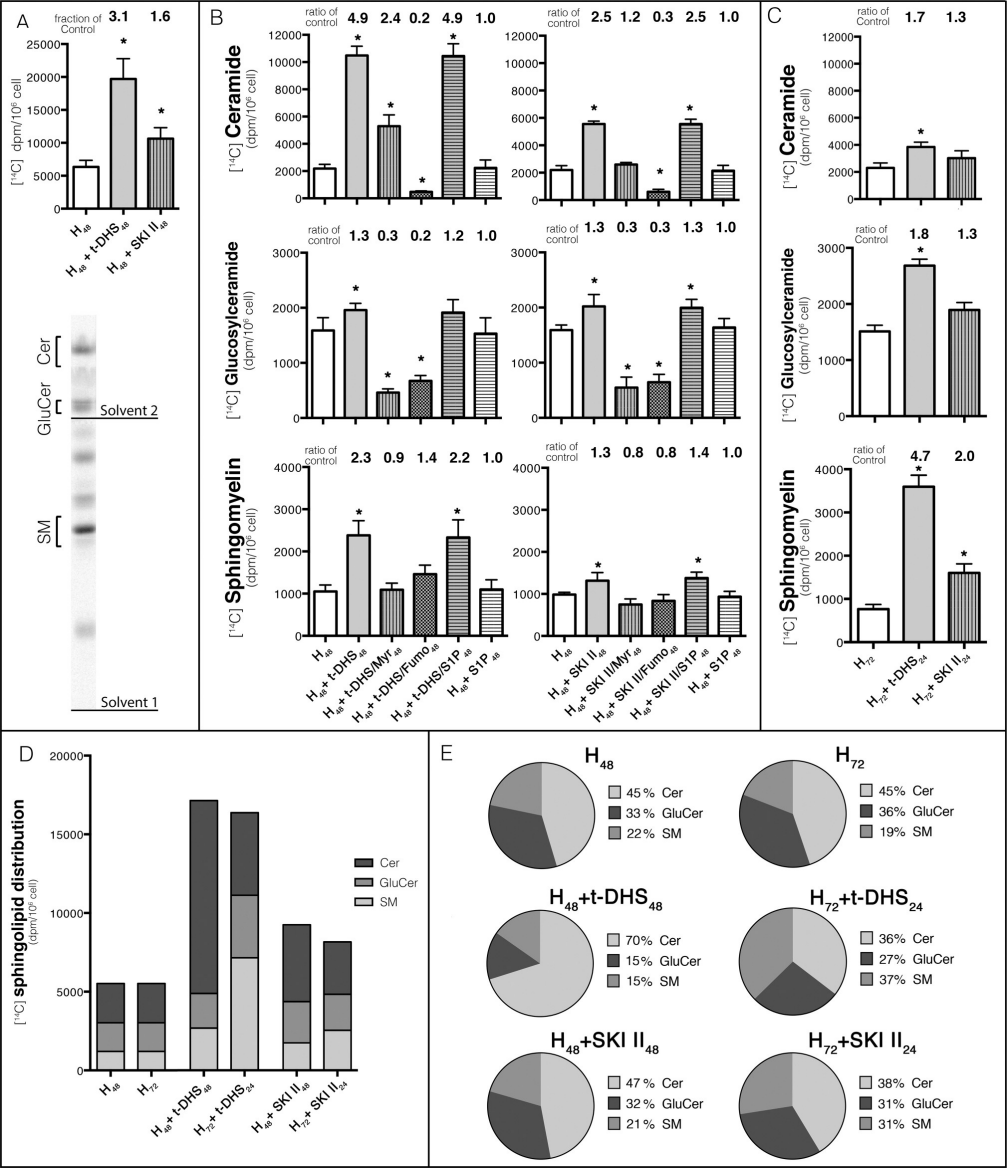
SK inhibition induces an increase in *de novo* sphingolipid synthesis. MDCK cells were labeled with [^14^C] palmitic acid for 4 hours before trypsinization. Sphingolipids were developed in two solvent systems: first in butanol:acetic acid:water (60:20:20, v/v/v; solvent 1), and the top portion of the TLC was run in solvent 2 (chloroform:methanol, 50:1.5, v/v) and detected by autoradiograph. The radioactivity associated with the cells and culture medium was quantified using liquid scintillation for all spots. (A) Total [^14^C] palmitic acid incorporation in H_48_ and t-DHS- and SKI II-treated cells (above), representative TLC plate (below). (B) Incorporation of [^14^C] palmitic acid to Cer, GluCer and SM in cells subjected to hypertonicity (H_48_) in the presence of t-DHS or SKI II and combination with Myr, Fumo and S1P. (C) [^14^C] palmitic acid incorporation to Cer, GluCer and SM in cells that were differentiated for 48 hours in the presence of hypertonicity and then treated with t-DHS or SKI II for 24 additional hours under hypertonicity (H_72_, H_72_+t-DHS_24_, H_72_+SKI II_24_). (D) Total and (E) relative [^14^C] palmitic incorporation to Cer, GluCer and SM are shown for the different conditions. Data are given as mean ± SD with n = 3, *p < 0.05.

The above results showed that SK inhibition causes deleterious effects in cells in progress to differentiation but not in already differentiated cells. On the other hand, we also showed that such effect was prevented when cells were treated with inhibitors of sphingolipid biosynthesis. To obtain a biochemical explanation of this phenomenon, we next studied sphingolipid synthesis in cells in progress to differentiation and differentiated cells in the presence or absence of the SK inhibitors. To this end, we used [^14^C] palmitic acid as a radioactive precursor, whose incorporation can occur either by *de novo* synthesis or by the salvage pathway. By using the one-dimension two-solvent system TLC, we can properly separate Cer from the final products SM, GluCer and LacCer. An autoradiography of a representative TLC plate is shown in Fig 7A. The quantitative analysis shows that cells in progress to differentiation, in response to treatment with SK inhibitors increased the incorporation of [^14^C] palmitic acid associated with total sphingolipids (Fig 7A). The analysis of the individual sphingolipids, whose identity was determined by mass spectrometry [16], showed that the treatment of cells with t-DHS evoked an increase in the radioactivity associated with Cer (4.9-fold). A small but significant increase was observed in both GluCer (1.3-fold) and SM (2.3-fold). Although lower, the same pattern was obtained by using SKI II. To evaluate whether the increase in sphingolipid synthesis involved *de novo* synthesis, Myr, a SPT inhibitor, was added simultaneously with the SK inhibitors. The treatment with Myr over SK inhibitors evoked a decrease of radioactivity associated to Cer, GluCer and SM. However, the level of radioactivity associated with Cer and SM was not lower than the control cells (H_48_), suggesting that Cer could be partially resynthesized by the salvage pathway. In turn, 70% of inhibition was obtained in GluCer in cells treated with t-DHS + Myr, reflecting that most of the GluCer is formed by *de novo* synthesis. In the case of SM, the inhibition of SPT blocked only the increase induced by SK inhibition. Alternatively, Fumo, a CerS/DHCerS inhibitor, was added to block the ceramide accumulation. Cells treated with t-DHS + Fumo showed a more profound inhibition of Cer synthesis, while the inhibition of GluCer and SM was similar to that induced by Myr.

The metabolic results suggest the coexistence of the *de novo* and salvage pathways of sphingolipid in MDCK cells in progress to differentiation, and that the increase induced by the SK inhibitors is mostly due to stimulation of SPT at least for Cer and SM, the most affected sphingolipid metabolites.

Considering that many cell types can release S1P and activate signaling pathways through its specific receptors (S1PR), SK inhibition was combined to exogenous S1P addition to rule out this possibility. As seen in Fig 7B, the exogenously added S1P was not able to reverse the increase in sphingolipid synthesis evoked by SK inhibitors, which shows that the regulation of sphingolipid synthesis is exerted by the intracellular and not the extracellular level of S1P. Additionally, the incubation with only S1P did not modify the sphingolipid *de novo* synthesis (Fig 7B). Moreover, the incubation with S1PR1 or S1PR2 antagonist (W146 or JTE-013, respectively) did not modify the metabolism either (S2 Fig).

Considering that FBS contains S1P and that this could affect sphingolipid metabolism, we studied the sphingolipid *de novo* synthesis in cultured cells grown in medium containing S1P-depleted FBS. As seen in S2 Fig, no differences in the incorporation of [^14^C] palmitic acid into Cer, GluCer or SM were observed between S1P-depleted and non-depleted FBS (H_48_ non-depleted FBS and H_48_ S1P-depleted FBS). Similarly, the increase of [^14^C] palmitic acid incorporation into Cer, GluCer and SM evoked by SK inhibition was the same in S1P-depleted FBS and non-depleted FBS (H_48_+t-DHS_48_/SKI II_48_ non-depleted FBS and H_48_+t-DHS_48_/SKI II_48_ S1P-depleted FBS). Moreover, exogenous S1P addition did not reverse the effects of SK inhibitors on the SLP metabolism. These results showed that the intracellular level of S1P, but not the extracellular one, modulates sphingolipid metabolism.

We further studied the metabolism in already differentiated cells. The pattern of distribution of [^14^C] palmitic acid in the various sphingolipid metabolites was similar to that of cells in progress to differentiation (Fig 7C). However, when cells were treated with t-DHS, the change in Cer was 1.7-fold, in GluCer 1.8-fold and in SM 4.7-fold. The same pattern, although attenuated, was obtained by using SKI II as a SK inhibitor. However, it is evident that while the change in radioactivity associated with Cer induced by t-DHS was only 1.7- vs the 4.9-fold of the Cer in cells in progress to differentiation, the opposite occurred with SM (2.3-fold vs 4.7-fold, respectively).

For a better comparison between the two experimental conditions, Fig 7D shows the total [^14^C] palmitic acid incorporation in the various sphingolipids in cells in progress to differentiation (H_48_ and SK inhibitors) and in already differentiated cells (H_72_ and SK inhibitors). Besides, Fig 7E shows the relative incorporation of [^14^C] palmitic acid to Cer, GluCer and SM in pie charts. Whereas cells with SK inhibited during the cell differentiation process mainly accumulated Cer (with 71% for t-DHS and 47% for SKI II) instead of SM (15% for t-DHS and 21% for SKI II), cells which had differentiated before SK was inhibited showed greater accumulation of SM (37% for t-DHS and 31% for SKI II) and a reduced accumulation of Cer (36% for t-DHS and 38% for SKI II).

These results showed that sphingolipid *de novo* synthesis in MDCK cells is modulated by intracellular S1P, but the response is different according to the degree of cell differentiation. While cells in progress to differentiation show an increase in sphingolipid synthesis reflected mainly in Cer accumulation, already differentiated cells branch the sphingolipid metabolism to SM and GluCer, thus evoking less accumulation of Cer. These results are consistent with our previous reports where we showed an overexpression of GluCer synthase and SM synthase in differentiated cells [14,15].

### 3.8. SK1 participates in MDCK cell differentiation

Different siRNA for SK1 or SK2 were used to assess the specificity of SK inhibitors and to dissect which SK isoforms are involved in the process.

**Fig 8:**
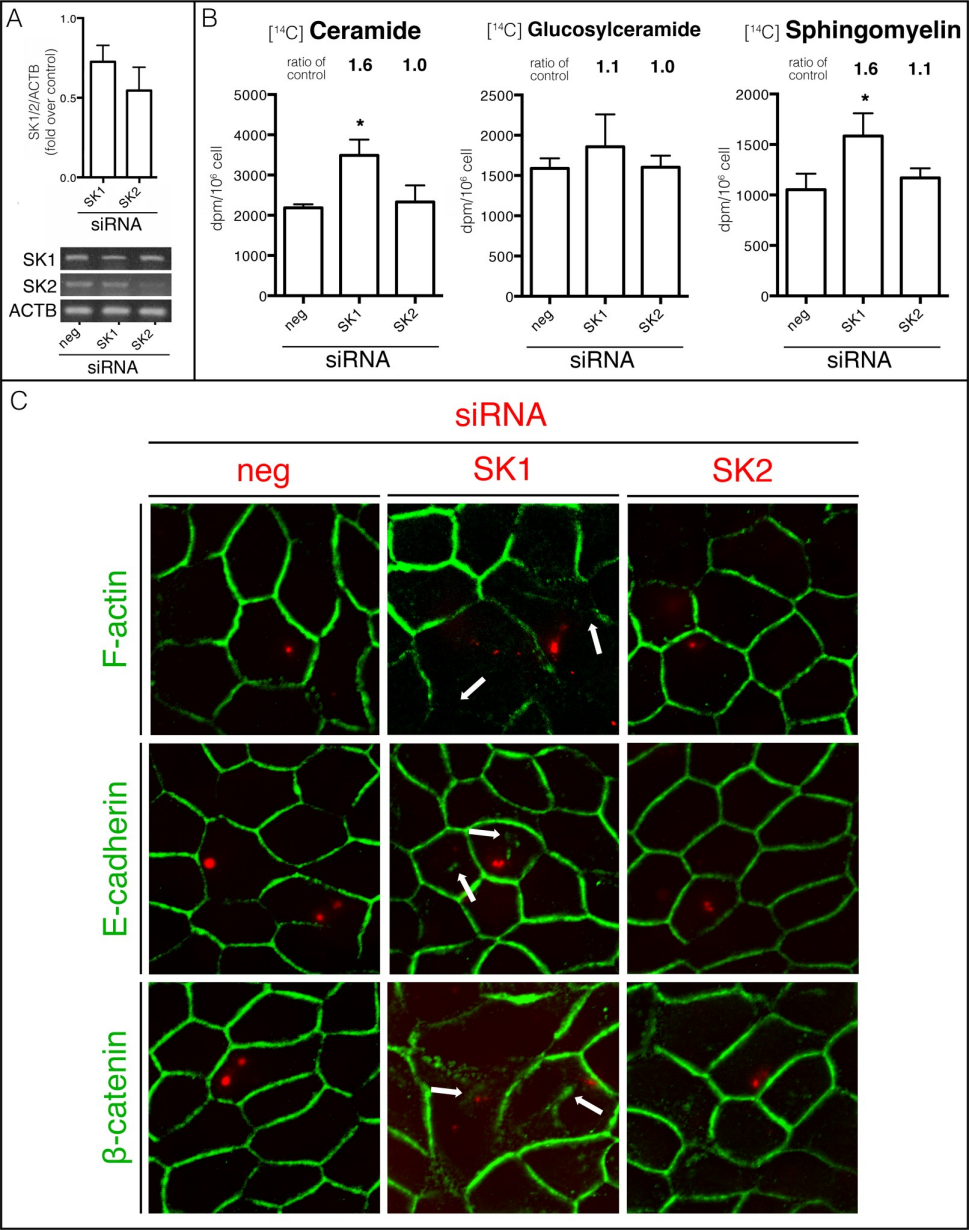
SK1 participates in MDCK cell differentiation. MDCK cells were transfected with 100 nM double-stranded siRNAs for SK1, SK2 or negative sequence. (A) SK1- or SK2-specific siRNA knockdown were checked by RT-PCR analysis (ACTB serves as internal control). (B) Cells were labeled with [^14^C] palmitic acid for 4 hours before trypsinization. Sphingolipids were developed by TLC and [^14^C] palmitic acid incorporation to Cer, GluCer and SM were quantified using liquid scintillation. (C) To identify transfected cells, monolayers were cotransfected with an Alexa546-labeled non-targeting siRNA whose signal is shown as red spots in the transfected cells. After treatment, fixed-cell monolayers were labeled for F-actin with phalloidin-FITC, E-cad and β-cat. Data are given as mean ± SD with n = 3, *p < 0.05.

The analysis of individual sphingolipids labeled with [^14^C] palmitic acid showed that the knockdown of SK1, but not SK2, evoked an increase in the radioactivity associated with Cer (1.6-fold) and SM (1.6-fold) compared with the negative siRNA control, but no significant increase in GluCer (Fig 8B). Although less pronounced, the same pattern was obtained in siRNA experiments and SK inhibitor assays, which confirms that the effects on sphingolipid metabolism observed in the pharmacological experiment were due to specific SK inhibition. The slighter increase in radioactivity associated with the different sphingolipids was due to the efficiency of transfection in our model (~30%). Interestingly, only SK1 knockdown was able to modulate total sphingolipid metabolism, but not SK2, which suggests that SK1 is the isoform involved in metabolism regulation.

After demonstrating that SK1 but not SK2 was able to control sphingolipid synthesis, cell morphology and tissue organization were evaluated by determining F-actin distribution using phalloidin-FITC as fluorochrome and performing fluorescence microscopy. In cells treated with negative siRNA (control), F-actin appeared delimiting the cells in transfected (red dotted cells) and non-transfected cells (Fig 8C F-actin). Next, we examined the effect of SK1 and SK2 knockdown on F-actin distribution. SK1 knockdown (red dotted cells) induced alterations in F-actin organization which impaired the cortex of F-actin drawing the cell limits (white arrow). Interestingly, SK2 knockdown did not alter F-actin distribution.

In order to confirm the importance of each SK isoform in AJ maturation, E-cadh and β-cat distribution was evaluated. As expected, in cells transfected with negative siRNA control (Fig 8B-neg, red dotted cells) both E-cad and β-cat showed appeared to delineate the cell periphery, thus reflecting the presence of mature AJs. In cells were SK1 was silenced (Fig 8B-SK1, red dotted cells), we observed the presence of intracellular accumulation of E-cad and β-cat (white arrowhead) as compared to non-transfected neighbouring cells, which suggests that SK1 knockdown altered AJ protein distribution. On the other hand, SK2 knowndown was unable to induce AJ protein intracellular accumulation, suggesting that SK2 is not involved in AJ maturation.

Taken together, knockdown experiments (in agreement with pharmacological inhibitor experiment) showed that alterations in sphingolipid metabolism affected MDCK cell differentiated morphology and impaired AJ complex distribution. Moreover, SK1, the isoform involved in sphingolipid metabolism modulation, but not SK2, was associated with differentiated cell morphology and AJ maturation, which reinforces the finding that *de novo* synthesis regulation by SK1/S1P is necessary for epithelial cell differentiation.

## 4. Discussion

Our biggest goal has been to define the role of sphingolipid metabolism in cellular fate. We have previously shown that, by modulating the expression and activity of sphingolipid metabolic enzymes, MDCK cells can direct the formation of the final metabolites that define cellular fate. In fact, we have reported that SK is highly expressed in proliferative state, thus maintaining cell proliferation and survival, while in differentiated cells, increased expression and activity of SM synthase and GluCer synthase ensure the establishment and preservation of cell-cell adhesion and mature apical membrane domain, typical of differentiated renal collecting duct cells [14–16].

The focus of the present study was to determine whether sphingolipid metabolism influences the process of differentiation and tissue organization of renal collecting duct cells. The results revealed that the SK1/S1P pathway, by modulating the *de novo* pathway of sphingolipid synthesis, is critical for MDCK differentiation and tissue organization but does not play a role when cells are already differentiated. The involvement of S1P in regulating the *de novo* biosynthesis of Cer has been long reported [36]. Thereafter, Tale et al. proposed the basal activity of SK as the most important factor for the accumulation of *de novo* formed Cer in breast cancer cells [37]. We further demonstrated that, in proliferating MDCK cells, the high level of SK expression and S1P endogenous level negatively control the *de novo* synthesis pathway by preventing Cer accumulation, which leads to the preponderance of S1P over Cer necessary for maintaining the proliferative state of cells [9]. We also demonstrated that the decrease in the endogenous levels of S1P evoked by SK inhibition triggers the *de novo* pathway with accumulation of Cer, thus causing cell death [9]. Based on this evidence, we hypothesized that, by controlling the *de novo* pathway of sphingolipid metabolism, basal SK/S1P could play a central role in determining cellular fate. Previously, by studying the sphingolipid content in the kidney of neonatal and adult rats, which represent non-differentiated/proliferating cells and fully differentiated/non-proliferating cells respectively, we found highest concentration of S1P in the neonatal period and decreased levels in the adult life, and Cer and SM increased contents in the adult [8,38]. To evaluate the physiological role of sphingolipid, in the present study, we emulated kidney development and maturation by using MDCK cells in different physiological stages: non-differentiated proliferating, polarized and differentiated cells. Consistent with the SK and S1P temporal gradient reported in developing and adult kidney, here we showed highest levels of SK and S1P in proliferating cells, decreasing levels in polarized cells, and lowest levels in differentiated cells (Fig 1). We also found that proliferating and polarized MDCK cells were highly sensitive to the endogenous levels of S1P and that, as cells mature, they become more resistant to endogenous S1P, suggesting that the survival of differentiated cells is independent of the endogenous levels of S1P (Fig 1). However, even in differentiated cells, the endogenous levels of S1P control the *de novo* synthesis pathway, as reflected by the increase in the radioactivity associated with the various sphingolipid metabolites when endogenous S1P synthesis was inhibited (Fig 7). Interestingly, the results using S1P-depleted medium showed that S1P modulates the sphingolipid metabolism by an intracellular mechanism and that S1P receptors are not involved (S2 Fig). Then, we were interested in elucidating the role, if any, of the regulation of sphingolipid metabolism exerted by S1P levels in cells in process of differentiation. Under basal conditions, the distribution of the radioactivity among the various metabolites in cells in process of differentiation and already differentiated cells was almost equal. However, the triggering of the *de novo* pathway evoked by SK inhibition resulted in higher accumulation of Cer in non-differentiated cells, while differentiated cells directed the metabolic pathway to the formation of SM and GluCer. This result is consistent with our previous observation that the activity and expression of both SM and GluCer synthases are increased in differentiated MDCK cells. This increased formation of sphingolipid metabolites resulted in the draining of *de novo* formed Cer, thus buffering its intracellular accumulation, which finally prevents its deleterious effects. The inhibition of SK in cells in progress to differentiation impaired the acquisition of the differentiated phenotype of MDCK cells and affected the adhesion to neighboring cells (Figs 2 and 3). It is known that the assembly of E-cad-based AJs is obligatory for the establishment of cell-cell adhesion [39]. Two major steps have to occur for the establishment of mature cell-cell adhesion: the formation of the E-cad/ β-cat/α-cat complex and its trafficking to the plasma membrane. The mechanism of the cadherin/catenins complex assembly and the targeting of E-cad to the basolateral membrane domain have been intensively studied recently. It has been suggested that β-cat binds E-cad co-translationally, but that α-cat binds after the complex has been transported to the plasma membrane [40,41]. However, more recent evidence supports the idea that β-cat and α-cat form a complex in the cytosol and, thereafter, the complex must be transported to the plasma membrane, where linkage to the actin cytoskeleton occurs [42,43]. Our experiments indicate that inhibition of SK does not seem to interfere with the assembly of the E-cad/β-cat/α-cat complex but that the complex remains in the TGN (Fig 4), secondary to a disbalance in the sphingolipid *de novo* synthesis, which causes Cer accumulation. This idea is supported by two observations. First, the AJ complex dissipated from the Golgi when *de novo* Cer synthesis was inhibited and, concomitantly, the cellular phenotype was rescued (Fig 5). We hypothesized that cells in progress to differentiation are unable to drain sufficiently the excess of *de novo* formed Cer which accumulates in the TGN. Thereafter, Cer accumulation probably generates a highly hydrophobic lipid domain, thus sequestering the AJ complex, probably blocking its vesicular traffic to the plasma membrane. Since the half-life of E-cad is rather short [44,45], retention of E-cad in the TGN would result in reduced cell surface expression of E-cad and consequently in the impairment of cell-cell adhesion. In contrast to cells in differentiation, differentiated cells were able to prevent E-cad/catenins retention in the TGN, thus preserving cell-cell adhesion (Fig 5). This was probably because they are more efficient to drain the *de novo* formed Cer.

It is accepted that E-cad is able to form a complex with catenins linked to the actin cytoskeleton, allowing the formation of the actin cortex, which is necessary to maintain the typical epithelial cell shape as seen in differentiated MDCK cells (Fig 2). The inhibition of SK induced a remodeling of the actin cytoskeleton all over the cell area. These effects caused changes in the cell morphology and cellular phenotype commonly observed in the EMT.

It is accepted that the loss of cell adhesion is a key step of the EMT. Mesenchymal transition in kidney collecting duct cells associated with disruption of E-cad/catenin-based cell adhesion has been previously reported [46]. Ivanova et al. demonstrated the ability of renal collecting duct cells to undergo EMT by disruption of the plasma membrane E-cad/catenin complex when stimulated by TGFβ [46]. Cadherin-based adhesion is not a static condition but rather a dynamic equilibrium between the cadherin complex at AJs and those in intracellular vesicles and compartments [47,48]. The pool of intracellular vesicles is composed of vesicles that come from endocytosis or from the TGN sorting to the plasma membrane [49]. Adhesion and endocytosis are closely opposed processes and disruption of the E-cad-based cell adhesion is commonly associated with an abuse of endocytosis over adhesion [47]. However, it is also considered that the proper sorting of E-cad and the efficient formation of vesicles are crucial steps for maintaining cadherin tracking and its cellular function. Delivery and turnover of E-cad at the plasma membrane provide a ready-made mechanism for cells to modulate cell adhesion depending on the adhesion requirements. Le et al. demonstrated that, in confluent polarized MDCK cells, a small amount of surface E-cad recycles to and from the plasma membrane, even in the absence of exogenous experimental manipulation [47,48]. Our results showed that the disbalance of the *de novo* formed Cer induced by SK inhibition affects the steps of the formation of the E-cad/catenin-containing vesicles directed to adhesion. Considering that a portion of the internalized cadherin can also enter late endosomes to be degraded and that the engagement of the vesicle trafficking machinery is deficient, cells in the process of differentiation may not accomplish the increased adhesion and consequently undergo EMT instead of differentiating.

The EMT is commonly accompanied by the nuclear translocation of the transcription factor Snail, which promotes EMT during development [50]. The Snail superfamily of transcription factors represses the transcription of E-cad, thus emerging as key regulators in the EMT process [51]. Our experiments showed that nuclear translocation of Snail I and II was secondary to Cer accumulation since, when *de novo* Cer synthesis was inhibited, Snail nuclear accumulation dissipated (Fig 6). This suggests that the signal for Snail translocation occurs at the time when plasma membrane E-cad diminishes and that when E-cad-based cell adhesion is re-established, Snail I and II dissipate from the nucleus. This result is consistent with the previous observation of Ivana et al., who described that disruption of E-cad/catenin-based adhesion together with SNAIL nuclear translocation are both early events in the EMT [46]. Although controversial with the commonly accepted concept that SNAIL nuclear translocation precedes E-cad/catenin-based cell adhesion disruption, our results showed that both phenomena seem to occur instantly and both stem from the accumulation of *de novo* Cer.

Interestingly, by performing SK1 or SK2 knockdown experiments we demonstrated that SK1, but not SK2, is the isoform involved in the regulation of sphingolipid metabolism and the consequent acquisition of a differentiated phenotype, thus reinforcing the concept that SK1 and SK2 can have different physiological implications [52,53].

In conclusion, the results of this work show additional evidence about the role of sphingolipid metabolism in cellular fate, emphasizing the role of SK/S1P pathway in the regulation of Cer *de novo* synthesis. We suggest that the expression and activity of SK act as a control system, allowing the cells to synchronize the various branches of sphingolipid metabolism to adequately respond to the cellular demand.

Further important evidence that emerges from these results is the high resistance of differentiated cells to the low levels of S1P, in contrast with proliferating cells or cells that suffered EMT. Taking into consideration that S1P is considered an oncometabolite because of its high expression in many cancer cells, our evidence about the different susceptibility of cells to SK inhibition could be of high importance for the therapy of cancer, since at concentrations of SK inhibitors that are lethal for cancer cells, normal cells probably survive.

## Supporting information

S1 Fig Effect of S1P-depleted FBS on cell morphology. The acquisition of the differentiated phenotype was analyzed by β-cat and F-actin distribution in cells cultured with S1P-depleted or non-depleted FBS. Cells were subjected to hypertonicity and treated with SK inhibitors (H_48_+t-DHS_48_ or H_48_+SKI II_48_) and 10 μM S1P (H_48_+t-DHS/S1P_48_ or H_48_+SKI II/S1P_48_).
(TIF)

S2 Fig Effect of S1P-depleted FBS on sphingolipid metabolism. Radiolabeled precursor incorporation in cells cultured with S1P-depleted vs non-depleted FBS. MDCK cells were incubated with S1P-depleted or non-depleted FBS and labeled with [^14^C] palmitic acid for 4 h before trypsinization. Sphingolipids were resolved as described previously. Graphs show the incorporation of [^14^C] palmitic acid to Cer, GluCer and SM in cells subjected to hypertonicity (H_48_) in the presence of t-DHS, SKI II W146 or JTE-013 (H_48_+t-DHS_48_, H_48_+t-SKI II_48_, H_48_+10μM W146 or H_48_+10μM JTE-013) and with addition of exogenous 10 μM S1P (H_48_+t-DHS/S1P_48_ or H_48_+SKI II /S1P_48_). Data are given as mean ± SD with n = 3, *p < 0.05 vs S1P non-depleted control (non-depleted-H_48_) or ˆp < 0.05 vs S1P-depleted control (S1P-depleted-H_48_).
(TIF)

S3 Fig Uncropped Western blots. The figure shows the original uncropped and unadjusted blots corresponding to (A) Fig 1, SK1 and actin and (B) Fig 3, E-cad. Bands in the E-cad blot correspond to E-cadherin (120/80 kDa) and E-cadherin precursor (135 kDa), according to manufacturer’s datasheet. In Fig 1, a mature E-cadherin band (~120 kDa) has been shown. The 35 kDa band could correspond to cleavage E-cadherin (35 kDa).
(TIF)

## Abbreviations

α-cat: α-catenin
β-cat: β-catenin
AJ: adherens Junction
Cer: ceramide
Con A: concavalin A
DHCer: dihydroceramide
E-cad: E-cadherin
EMT: Epithelial Mesenchymal transition
FBS: fetal bovine serum
GalT2: UDP-Gal:GA2/GM2/GD2 β-1,3-galactosyltransferase
GluCer: glucosylceramide
Fumo: Fumonisin B1
MDCK: Madin-Darby Canine Kidney
MOC: Manders' Overlap Coefficient
Myr: myriocin
S1P: sphingosine-1-phosphate
SK: sphingosine kinase
SM: sphingomyelin
SPT: Serine palmitoyl-CoA transferase
t-DHS: L-threo-dihydrosphingosine
TGN: trans-Golgi network

## References

[1] Bartke N, Hannun YA. Bioactive sphingolipids: metabolism and function. J Lipid Res. American Society for Biochemistry and Molecular Biology; 2009;50: S91–S96. 10.1194/jlr.R800080-JLR200 19017611 PMC2674734

[2] van Echten G, Birk R, Brenner-Weiss G, Schmidt RR, Sandhoff K. Modulation of sphingolipid biosynthesis in primary cultured neurons by long chain bases. J Biol Chem. United States; 1990;265: 9333–9339. 2111818

[3] Merrill AHJ. Sphingolipid and glycosphingolipid metabolic pathways in the era of sphingolipidomics. Chem Rev. United States; 2011;111: 6387–6422. 10.1021/cr2002917 21942574 PMC3191729

[4] Hanada K. Serine palmitoyltransferase, a key enzyme of sphingolipid metabolism. Biochim Biophys Acta. Netherlands; 2003;1632: 16–30. 12782147 10.1016/s1388-1981(03)00059-3

[5] Hannun YA, Obeid LM. Principles of bioactive lipid signalling: lessons from sphingolipids. Nature Publishing Group; 2008;9: 139 10.1038/nrm2329 18216770

[6] Proia RL, Hla T. Emerging biology of sphingosine-1-phosphate: its role in pathogenesis and therapy. J Clin Invest. United States; 2015;125: 1379–1387. 10.1172/JCI76369 25831442 PMC4409021

[7] Blaho VA, Hla T. An update on the biology of sphingosine 1-phosphate receptors. J Lipid Res. United States; 2014;55: 1596–1608. 10.1194/jlr.R046300 24459205 PMC4109755

[8] Facchinetti MM, Beuret C, Marquez MG, Sterin-Speziale N. Differential branching of the sphingolipid metabolic pathways with the stage of development. Involvement of sphingosine kinase. Biol Neonate. 2003/09/25. 2003;84: 243–251. 10.1159/000072308 14504448

[9] Nieto FL, Pescio LG, Favale NO, Adamo AM, Sterin-Speziale NB. Sphingolipid Metabolism Is a Crucial Determinant of Cellular Fate in Nonstimulated Proliferating Madin-Darby Canine Kidney (MDCK) Cells. J Biol Chem. 2008;283: 25682–25691. 10.1074/jbc.M804437200 18625703

[10] The subcellular organization of Madin-Darby canine kidney cells during the formation of a polarized epithelium. The Journal of Cell Biology. 1989 pp. 2817–2832. 2592406 10.1083/jcb.109.6.2817 PMC2115929

[11] Minuth WW, Steiner P, Strehl R, Schumacher K, de Vries U, Kloth S. Modulation of cell differentiation in perfusion culture. Exp Nephrol. Switzerland; 1999;7: 394–406. 10.1159/000020637 10559637

[12] Steiner P, Strehl R, Kloth S, Tauc M, Minuth WW. In vitro development and preservation of specific features of collecting duct epithelial cells from embryonic rabbit kidney are regulated by the electrolyte environment. Differentiation. England; 1997;62: 193–202. 9503604 10.1046/j.1432-0436.1998.6240193.x

[13] Matsuzaki T, Suzuki T, Takata K. Hypertonicity-induced expression of aquaporin 3 in MDCK cells. Am J Physiol Cell Physiol. United States; 2001;281: C55–63. 10.1152/ajpcell.2001.281.1.C55 11401827

[14] Pescio LG, Favale NO, Marquez MG, Sterin-Speziale NB. Glycosphingolipid synthesis is essential for MDCK cell differentiation. Biochim Biophys Acta. 2012/03/06. 2012;1821: 884–894. 10.1016/j.bbalip.2012.02.009 22387616

[15] Favale NO, Santacreu BJ, Pescio LG, Marquez MG, Sterin-Speziale NB. Sphingomyelin metabolism is involved in the differentiation of MDCK cells induced by environmental hypertonicity. J Lipid Res. 2015/02/12. 2015;56: 786–800. 10.1194/jlr.M050781 25670801 PMC4373737

[16] Pescio LG, Santacreu BJ, Lopez VG, Pavan CH, Romero DJ, Favale NO, et al Changes in ceramide metabolism are essential in Madin-Darby canine kidney cell differentiation. J Lipid Res. 2017/05/19. 2017;58: 1428–1438. 10.1194/jlr.M076349 28515139 PMC5496039

[17] Hinck L, Nathke IS, Papkoff J, Nelson WJ. Dynamics of cadherin/catenin complex formation: novel protein interactions and pathways of complex assembly. J Cell Biol. United States; 1994;125: 1327–1340. 8207061 10.1083/jcb.125.6.1327 PMC2290923

[18] Ozawa M, Kemler R. Molecular organization of the uvomorulin-catenin complex. J Cell Biol. United States; 1992;116: 989–996. 1734027 10.1083/jcb.116.4.989 PMC2289345

[19] Segal JM, Ward CM. Novel peptides for deciphering structural and signalling functions of E-cadherin in mouse embryonic stem cells. Sci Rep. England; 2017;7: 41827 10.1038/srep41827 28169326 PMC5294416

[20] Mohamet L, Hawkins K, Ward CM. Loss of function of e-cadherin in embryonic stem cells and the relevance to models of tumorigenesis. J Oncol. Egypt; 2011;2011: 352616 10.1155/2011/352616 21197469 PMC3005858

[21] Wang Y, Shi J, Chai K, Ying X, Zhou BP. The Role of Snail in EMT and Tumorigenesis. Curr Cancer Drug Targets. Netherlands; 2013;13: 963–972. 24168186 10.2174/15680096113136660102 PMC4004763

[22] Pan Y, Li J, Zhang Y, Wang N, Liang H, Liu Y, et al Slug-upregulated miR-221 promotes breast cancer progression through suppressing E-cadherin expression. Scientific Reports. 2016 10.1038/srep25798 27174021 PMC4865839

[23] Mikami S, Katsube K-I, Oya M, Ishida M, Kosaka T, Mizuno R, et al Expression of Snail and Slug in renal cell carcinoma: E-cadherin repressor Snail is associated with cancer invasion and prognosis. Lab Invest. United States; 2011;91: 1443–1458. 10.1038/labinvest.2011.111 21808237

[24] Cham BE, Knowles BR. A solvent system for delipidation of plasma or serum without protein precipitation. J Lipid Res. United States; 1976;17: 176–181. 818332

[25] Signorelli P, Hannun YA. Analysis and quantitation of ceramide. Methods Enzymol. United States; 2002;345: 275–294. 11665612 10.1016/s0076-6879(02)45023-9

[26] Bligh EG, Dyer WJ. A RAPID METHOD OF TOTAL LIPID EXTRACTION AND PURIFICATION. Can J Biochem Physiol. NRC Research Press; 1959;37: 911–917. 10.1139/o59-099 13671378

[27] Giraudo CG, Daniotti JL, Maccioni HJ. Physical and functional association of glycolipid N-acetyl-galactosaminyl and galactosyl transferases in the Golgi apparatus. Proc Natl Acad Sci U S A. 2001/01/23. The National Academy of Sciences; 2001;98: 1625–1630. 10.1073/pnas.98.4.1625 11172001 PMC29307

[28] Nyquist H. Certain Topics in Telegraph Transmission Theory. Trans Am Inst Electr Eng. 1928;47: 617–644. 10.1109/T-AIEE.1928.5055024

[29] Gao P, Peterson YK, Smith RA, Smith CD. Characterization of isoenzyme-selective inhibitors of human sphingosine kinases. PLoS One. United States; 2012;7: e44543 10.1371/journal.pone.0044543 22970244 PMC3438171

[30] Modrak DE, Gold D V, Goldenberg DM. Sphingolipid targets in cancer therapy. Mol Cancer Ther. 2006;5: 200 LP–208. 16505092 10.1158/1535-7163.MCT-05-0420

[31] Samak G, Gangwar R, Crosby LM, Desai LP, Wilhelm K, Waters CM, et al Cyclic stretch disrupts apical junctional complexes in Caco-2 cell monolayers by a JNK-2-, c-Src-, and MLCK-dependent mechanism. American Journal of Physiology—Gastrointestinal and Liver Physiology. Bethesda, MD; 2014 pp. G947–58. 10.1152/ajpgi.00396.2013 24722904 PMC4042113

[32] Zinchuk V, Zinchuk O, Okada T. Quantitative colocalization analysis of multicolor confocal immunofluorescence microscopy images: pushing pixels to explore biological phenomena. Acta Histochem Cytochem. Japan; 2007;40: 101–111. 10.1267/ahc.07002 17898874 PMC1993886

[33] Sheng L, Zhang S, Xu H. Effect of Slug-Mediated Down-Regulation of E-Cadherin on Invasiveness and Metastasis of Anaplastic Thyroid Cancer Cells. Med Sci Monit. United States; 2017;23: 138–143. 10.12659/MSM.902725 28070118 PMC5242203

[34] Yu Q, Zhang K, Wang X, Liu X, Zhang Z. Expression of transcription factors snail, slug, and twist in human bladder carcinoma. J Exp Clin Cancer Res. England; 2010;29: 119 10.1186/1756-9966-29-119 20809941 PMC2942802

[35] Medici D, Hay ED, Olsen BR. Snail and Slug promote epithelial-mesenchymal transition through beta-catenin-T-cell factor-4-dependent expression of transforming growth factor-beta3. Mol Biol Cell. United States; 2008;19: 4875–4887. 10.1091/mbc.E08-05-0506 18799618 PMC2575183

[36] van Echten-Deckert G, Zschoche A, Bär T, Schmidt RR, Raths A, Heinemann T, et al cis-4-Methylsphingosine Decreases Sphingolipid Biosynthesis by Specifically Interfering with Serine Palmitoyltransferase Activity in Primary Cultured Neurons. J Biol Chem. 1997;272: 15825–15833. 10.1074/jbc.272.25.15825 9188480

[37] Taha TA, Kitatani K, El-Alwani M, Bielawski J, Hannun YA, Obeid LM. Loss of sphingosine kinase-1 activates the intrinsic pathway of programmed cell death: modulation of sphingolipid levels and the induction of apoptosis. Faseb j. 2006/03/02. 2006;20: 482–484. 10.1096/fj.05-4412fje 16507765

[38] Facchinetti MM, Leocata Nieto F, Marquez MG, Sterin-Speziale N. Stratification of sphingosine kinase-1 expression and activity in rat kidney. Cells Tissues Organs. 2008/06/17. 2008;188: 384–392. 10.1159/000139770 18552482

[39] Baum B, Georgiou M. Dynamics of adherens junctions in epithelial establishment, maintenance, and remodeling. J Cell Biol. The Rockefeller University Press; 2011;192: 907–917. 10.1083/jcb.201009141 21422226 PMC3063136

[40] Curtis MW, Johnson KR, Wheelock MJ. E-cadherin/catenin complexes are formed co-translationally in the endoplasmic reticulum/Golgi compartments. Cell Commun Adhes. 2008;15: 365–378. 10.1080/15419060802460748 18937087 PMC2742162

[41] Drees F, Pokutta S, Yamada S, Nelson WJ, Weis WI. α-Catenin Is a Molecular Switch that Binds E-Cadherin-β-Catenin and Regulates Actin-Filament Assembly. Cell. 2005;123: 903–915. 10.1016/j.cell.2005.09.021 16325583 PMC3369825

[42] Pokutta S, Drees F, Yamada S, Nelson WJ, Weis WI. Biochemical and structural analysis of α-catenin in cell–cell contacts. Biochem Soc Trans. 2008;36: 141–147. 10.1042/BST0360141 18363554 PMC3369830

[43] Pokutta S, Choi HJ, Ahlsen G, Hansen SD, Weis WI. Structural and thermodynamic characterization of cadherin.beta-catenin.alpha-catenin complex formation. J Biol Chem. 2014/04/03. 2014;289: 13589–13601. 10.1074/jbc.M114.554709 24692547 PMC4036364

[44] Chen Y-T, Stewart DB, Nelson WJ. Coupling Assembly of the E-Cadherin/β-Catenin Complex to Efficient Endoplasmic Reticulum Exit and Basal-lateral Membrane Targeting of E-Cadherin in Polarized MDCK Cells. J Cell Biol. The Rockefeller University Press; 1999;144: 687–699. 10037790 10.1083/jcb.144.4.687 PMC2132940

[45] Gumbiner BM. Regulation of Cadherin Adhesive Activity. J Cell Biol. The Rockefeller University Press; 2000;148: 399–404. 10662767 10.1083/jcb.148.3.399 PMC2174801

[46] Ivanova L, Butt MJ, Matsell DG. Mesenchymal transition in kidney collecting duct epithelial cells. Am J Physiol Ren Physiol. 2008/03/07. 2008;294: F1238–48. 10.1152/ajprenal.00326.2007 18322023

[47] Le TL, Yap AS, Stow JL. Recycling of E-cadherin: a potential mechanism for regulating cadherin dynamics. J Cell Biol. 1999/07/14. 1999;146: 219–232. 10402472 PMC2199726

[48] Xiao K, Allison DF, Buckley KM, Kottke MD, Vincent PA, Faundez V, et al Cellular levels of p120 catenin function as a set point for cadherin expression levels in microvascular endothelial cells. J Cell Biol. The Rockefeller University Press; 2003;163: 535–545. 10.1083/jcb.200306001 14610056 PMC2173638

[49] Yap AS, Crampton MS, Hardin J. Making and breaking contacts: The cellular biology of cadherin regulation. Curr Opin Cell Biol. 2007;19: 508–514. 10.1016/j.ceb.2007.09.008 17935963 PMC2128038

[50] Muqbil I, Wu J, Aboukameel A, Mohammad RM, Azmi AS. Snail Nuclear Transport: the Gateways Regulating Epithelial-to-Mesenchymal Transition? Semin Cancer Biol. 2014;27: 39–45. 10.1016/j.semcancer.2014.06.003 24954011 PMC4165636

[51] Batlle E, Sancho E, Franci C, Dominguez D, Monfar M, Baulida J, et al The transcription factor snail is a repressor of E-cadherin gene expression in epithelial tumour cells. Nat Cell Biol. 2000/02/03. 2000;2: 84–89. 10.1038/35000034 10655587

[52] Yuza K, Nakajima M, Nagahashi M, Tsuchida J, Hirose Y, Miura K, et al Different Roles of Sphingosine Kinase 1 and 2 in Pancreatic Cancer Progression. J Surg Res. United States; 2018;232: 186–194. 10.1016/j.jss.2018.06.019 30463717

[53] Alemany R, van Koppen CJ, Danneberg K, Ter Braak M, Meyer Zu Heringdorf D. Regulation and functional roles of sphingosine kinases. Naunyn Schmiedebergs Arch Pharmacol. 2007/01/24. 2007;374: 413–428. 10.1007/s00210-007-0132-3 17242884

