# Supplementary figures and images for: Sphingosine kinase and sphingosine-1-phosphate regulate epithelial cell architecture by the modulation of de novo sphingolipid synthesis
Source: PLoS One. 2019 Mar 21;14(3):e0213917. doi: 10.1371/journal.pone.0213917 (PMC6428330; doi:10.1371/journal.pone.0213917)

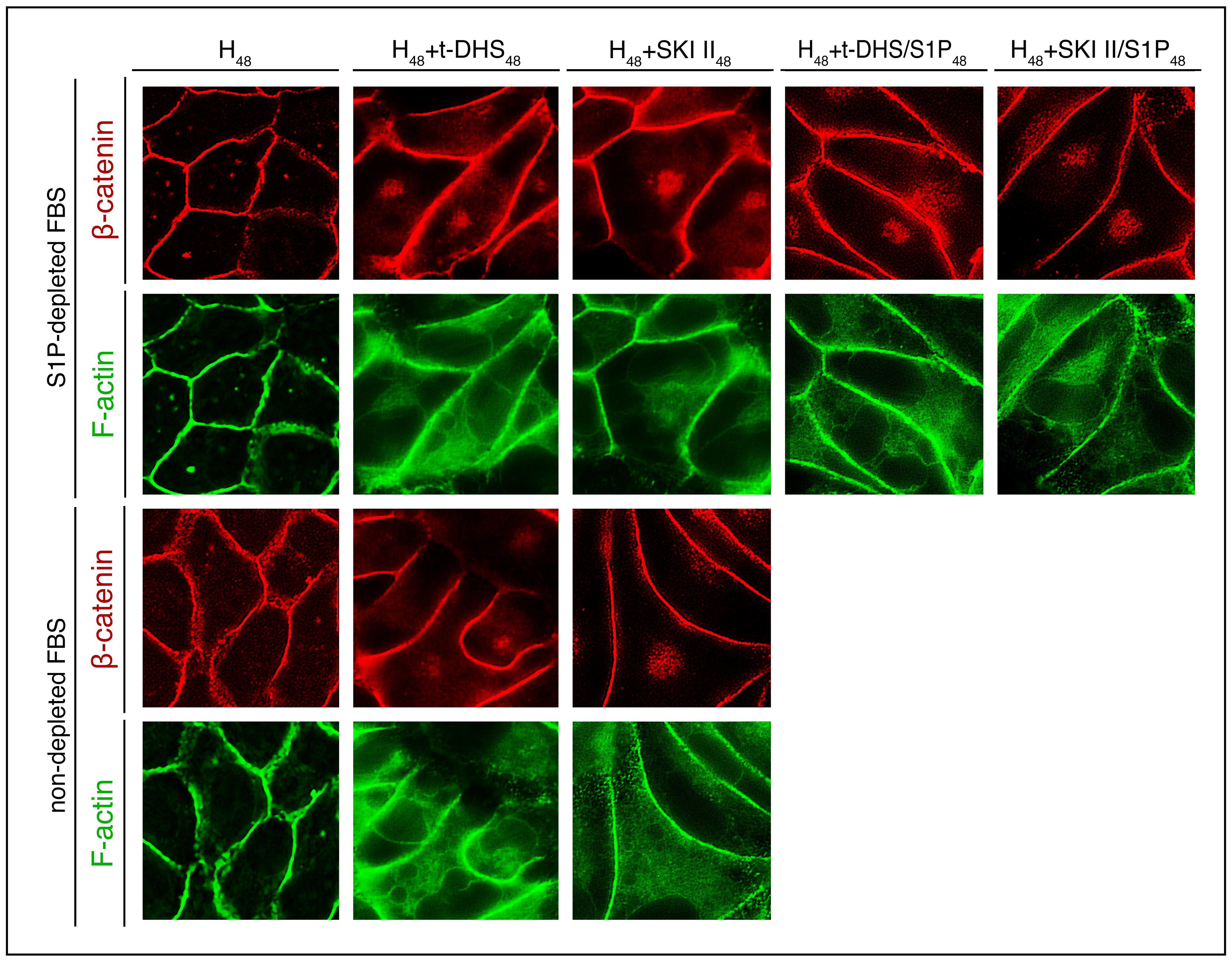

Supplement: S1 Fig — The acquisition of the differentiated phenotype was analyzed by β-cat and F-actin distribution in cells cultured with S1P-depleted or non-depleted FBS. Cells were subjected to hypertonicity and treated with SK inhibitors (H48+t-DHS48 or H48+SKI II48) and 10 μM S1P (H48+t-DHS/S1P48 or H48+SKI II/S1P48). (TIF) [file pone.0213917.s001.tif]

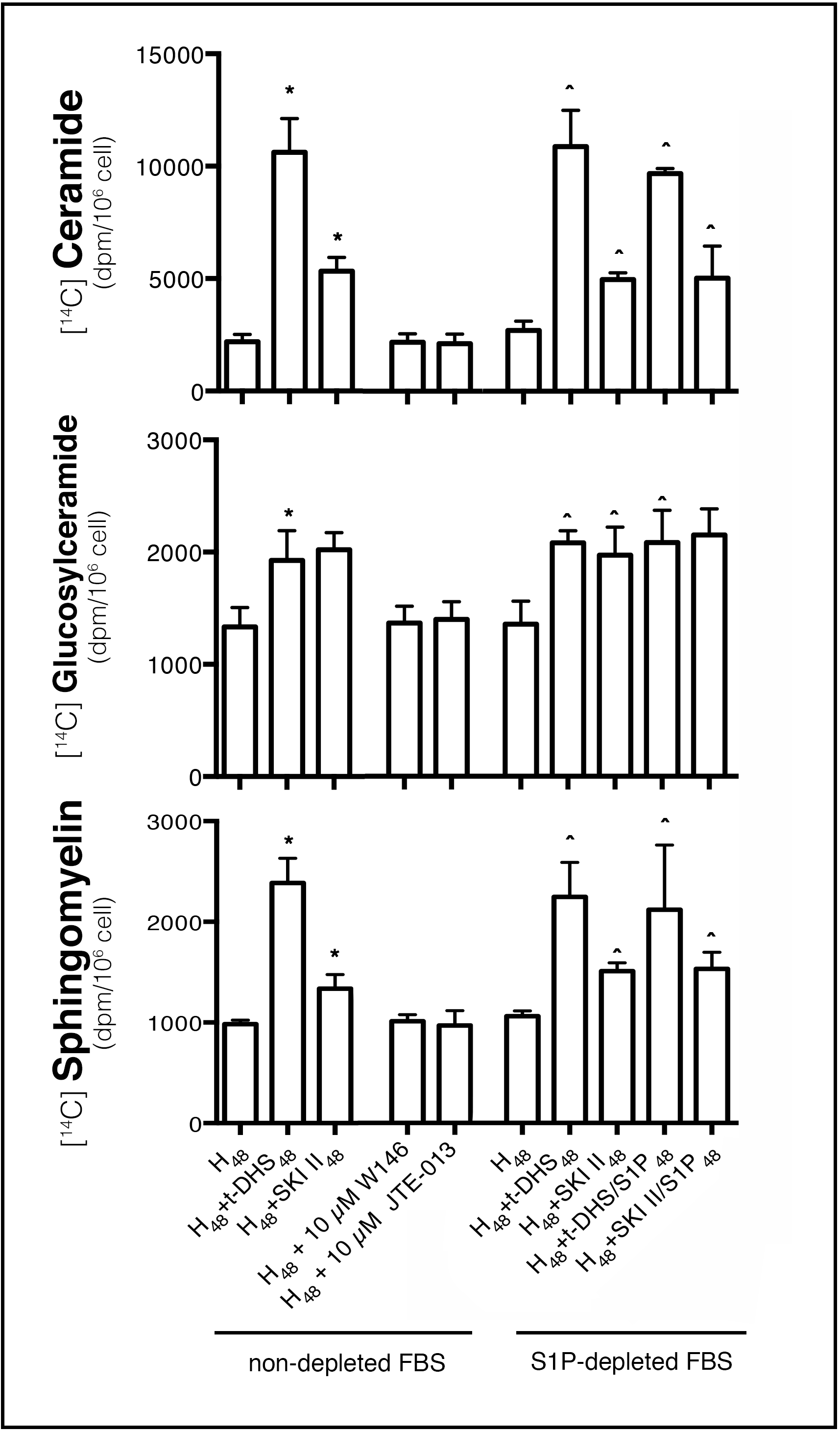

Supplement: S2 Fig — Radiolabeled precursor incorporation in cells cultured with S1P-depleted vs non-depleted FBS. MDCK cells were incubated with S1P-depleted or non-depleted FBS and labeled with [14C] palmitic acid for 4 h before trypsinization. Sphingolipids were resolved as described previously. Graphs show the incorporation of [14C] palmitic acid to Cer, GluCer and SM in cells subjected to hypertonicity (H48) in the presence of t-DHS, SKI II W146 or JTE-013 (H48+t-DHS48, H48+t-SKI II48, H48+10μM W146 or H48+10μM JTE-013) and with addition of exogenous 10 μM S1P (H48+t-DHS/S1P48 or H48+SKI II /S1P48). Data are given as mean ± SD with n = 3, *p < 0.05 vs S1P non-depleted control (non-depleted-H48) or ˆp < 0.05 vs S1P-depleted control (S1P-depleted-H48). (TIF) [file pone.0213917.s002.tif]

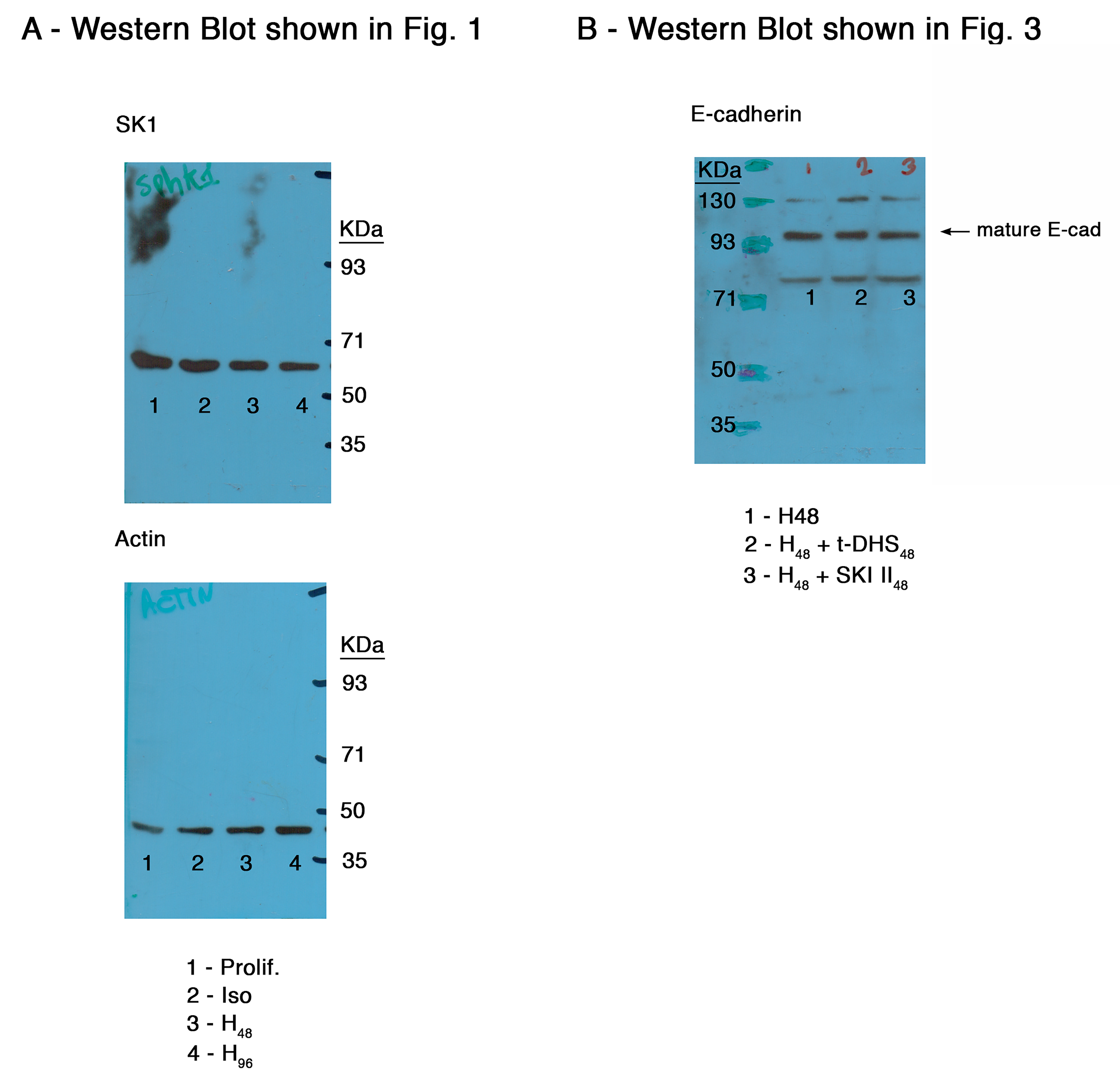

Supplement: S3 Fig — The figure shows the original uncropped and unadjusted blots corresponding to (A) Fig 1, SK1 and actin and (B) Fig 3, E-cad. Bands in the E-cad blot correspond to E-cadherin (120/80 kDa) and E-cadherin precursor (135 kDa), according to manufacturer’s datasheet. In Fig 1, a mature E-cadherin band (~120 kDa) has been shown. The 35 kDa band could correspond to cleavage E-cadherin (35 kDa). (TIF) [file pone.0213917.s003.tif]
